# A decision-space model explains context-specific decision-making

**DOI:** 10.21203/rs.3.rs-5499511/v1

**Published:** 2024-12-03

**Authors:** Dirk W. Beck, Cory N. Heaton, Luis D. Davila, Lara I. Rakocevic, Sabrina M. Drammis, Danil Tyulmankov, Paulina Vara, Atanu Giri, Shreeya Umashankar Beck, Qingyang Zhang, Michael Pokojovy, Kenichiro Negishi, Serina A. Batson, Alexis A. Salcido, Neftali F. Reyes, Andrea Y. Macias, Raquel J. Ibanez-Alcala, Safa B. Hossain, Graham L. Waller, Travis M. Moschak, Ki A. Goosens, Alexander Friedman

**Affiliations:** 1. Department of Biological Sciences, University of Texas at El Paso, El Paso, TX, USA; 2. Computational Science Program, University of Texas at El Paso, EI Paso, TX, USA; 3. Artificial Intelligence Laboratory, Department of Computer Science, Massachusetts Institute of Technology, Cambridge, MA, USA; 4. National Institute on Drug Abuse, Baltimore, MD, USA; 5. Ming Hsieh Department of Electrical and Computer Engineering, Viterbi School of Engineering, University of Southern California, Los Angeles, CA; 6. Department of Mathematics and Statistics, Old Dominion University, Norfolk, VA, USA; 7. Department of Psychiatry; Center for Translational Medicine and Pharmacology; Friedman Brain Institute; Icahn School of Medicine at Mount Sinai, NY, USA; 8. Lead author

## Abstract

Optimal decision-making requires consideration of internal and external contexts. Biased decision-making is a transdiagnostic symptom of neuropsychiatric disorders. We created a computational model demonstrating how the striosome compartment of the striatum constructs a context-dependent mathematical space for decision-making computations, and how the matrix compartment uses this space to define action value. The model explains multiple experimental results and unifies other theories like reward prediction error, roles of the direct versus indirect pathways, and roles of the striosome versus matrix, under one framework. We also found, through new analyses, that striosome and matrix neurons increase their synchrony during difficult tasks, caused by a necessary increase in dimensionality of the space. The model makes testable predictions about individual differences in disorder susceptibility, decision-making symptoms shared among neuropsychiatric disorders, and differences in neuropsychiatric disorder symptom presentation. The model provides new evidence for the central role that striosomes play in neuroeconomic and disorder-affected decision-making.

Decision-making is altered in neuropsychiatric disorders affecting the basal ganglia^1^. A range of experimental evidence links, in particular, balances between the compartments of the striatum and connected brain regions to decision-making function and dysfunction^2–6^. Understanding these intricate interactions will be crucial for designing next-generation treatments.

Striatal neurons can be categorized via neurochemistry and connectivity into groups, including the striosome and matrix compartments^4,7^. Striosomal spiny projection neurons (sSPNs) make up ~10–15% of the striatum and matrix spiny projection neurons (mSPNs) another ~85–90%^8,9^ (for acronyms, see Table 1). New technologies, including recording and targeting methods and genetically engineered mice, have enabled important new discoveries about differential roles for striosomes and matrix in decision-making^3,10–19^, including in disorders^2,4^. Further, both sSPNs and mSPNs belong to either the direct pathway (dsSPNs and dmSPNs), identified by D1 receptor expression, or the indirect pathway (dsSPNs and isSPNs) identified by D2 receptor expression^4,20^. Notably, dsSPNs, to a greater extent than dmSPNs, project in turn to regions which influence midbrain dopamine release via a subcircuit that is conserved across species^8,16,21–24^ (Fig. 1a, Table 2). Thus, dsSPNs, isSPNs, dmSPNs, and imSPNs appear to have different physiological roles^16,20^, raising the possibility they each play a distinct functional role during decision-making.

While a range of modeling works explore the roles of direct versus indirect pathway function^25^, they largely omit the important dimension of striosome versus matrix. The omission of striosomes from models of decision-making or basal ganglia function hinders the interpretation of important features of the striatum because it prevents an accurate depiction of striatal-daSNC interplay, which is primarily striosomal^4,24,26^ (Table 2). Further, disorders that differentially affect the direct versus indirect pathways have been found to also affect striosomes versus matrix differently^2^, suggesting that attention to all four compartments is necessary for an accurate understanding of disorder-affected decision-making. To close this gap, we formed a model that accounts for striosome versus matrix subdivisions, including the selective modulation of midbrain dopamine by striosomes (Fig. 1, Extended Data Fig. 1). From our physiological model arises the concept of a “decision-dimension,” our term for an axis along which the modeled circuit encodes information (for terminology, see Table 1). During a decision, decision-dimensions that are important based on the context are selected, forming a mathematical “decision-space.” We present evidence, via our analysis of the neural recordings and our models of findings from the experimental literature, for the core tenets of our model: that subpopulations of SPNs encode information along decision-dimensions, that a decision-space is formed, and that the decision-space adapts based on context (Figs. 2,3, Extended Data Figs. 2–5). Then we demonstrate the power of the model to explain a range of physiological and behavioral phenomena, including RPE and the roles of the indirect/direct pathway (Fig. 4, Extended Data Fig. 6). Finally, we speculate how the model might explain behavioral phenomena observed in psychiatry (Figs. 5,6, Extended Data Figs. 7,8) and suggest future experiments (Extended Data Fig. 9).

## Results

### Model description.

**Extended Data Fig. 1:**
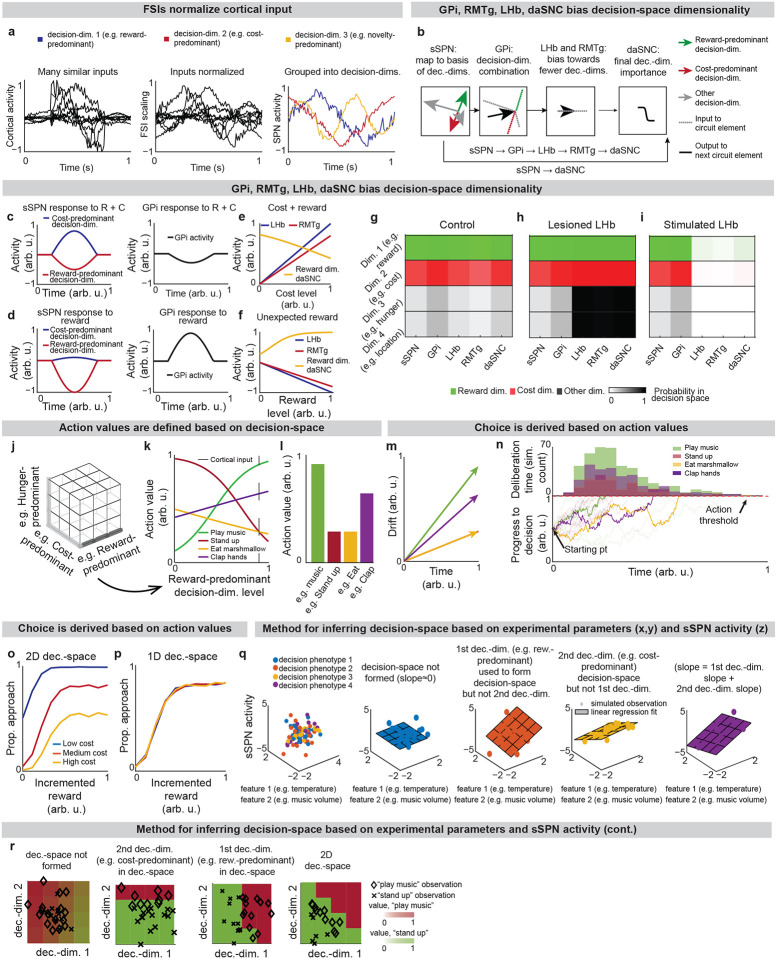
**Action values are defined within a decision-space formed by the circuit, Related to** Fig. 1. **a**, Example showing how FSIs and SPNs parse cortical activity in the model. The signals of 10 cortical neurons encoding sensory information (**a**) are normalized by FSIs such that activities are on a more uniform scale (**b**) and then mapped to sSPNs (**c**), which each encode activity along a “decision-dimension.” In this figure (and Fig. 1), an instance of the model is described which allows for two convenient simplifications: 1) the representation of SPNs (both sSPNs and mSPNs) using one circuit element per decision-dimension, and 2) the treatment of the circuit using a feedforward model. See ***Instance 1: full connectivity and feedforward,*** Methods. **b**, After FSI normalization, sSPNs map cortical activity to a basis of decision-dimensions. Mathematically, we represent, for a given pathway P (either direct pathway or indirect pathway), the mapping of cortical activity $x_{P}$ to decision-dimensions as a linear transform via the matrix $W_{P}$, whose columns correspond to the first several (in our analysis, 4) principal components of cortical activity. During this process, there is divisive normalization by FSI activity $c_{P}$ and the potential for an overall shift in SSPN activity $b_{\text{sSPN}}$ (eq. (1)). Next, GPi combines the sSPN signals into a single representation and RMTg and LHb bias this combined representation of signal along all decision-dimensions. We represent RMTg activity RMTg as the dot product of striosome to GPi weights $w_{\text{GPi}}$ and the activities the sSPNs $s_{\text{sSPN},P}$ for each pathway p, after incorporation of additive shifts $z_{\text{GPi}},z_{\text{LHb}}$, and $z_{\text{RMTg}}$ (eq. (5)). Next, daSNC takes input directly from sSPNs and from RMTg to calculate the final importance of each decision-dimension. We represent daSNC activity ${\text{daSNC}}_{\text{i},\text{P}}$ corresponding to decision-dimension i and pathway P as the activity of the corresponding sSPN population $s_{\text{sSPN},i,P}$ multiplied by a connection weight $w_{\text{sSPN}\to \text{daSNC},i,P}$, plus additive shifts applied individual to each daSNC element $\left(z_{\text{daSNC},i,P}\right)$ and uniformly to all daSNC elements (RMTg), all passed through an activation function (eq. (2)). **c,d,** Example where sSPNs parse reward and cost (**c**), or reward (**d**) inputs along decision-dimensions (left) and then GPi combines the signals along the decision-dimensions into a single representation (right). **e,f,** Modeled responses of LHb, RMTg, and daSNC to a cortical signal encoding reward and cost (**e**), and a cortical signal encoding an unexpected reward (**f**). LHb and RMTg increase their activities proportionally to the cost and decrease their activities proportionally to the reward. daSNC change their activities inversely. The modeled daSNC response is due to the combination of cortical inputs projected onto them directly from sSPN and through GPi, LHb, and RMTg. **g-I**, Roles of the circuit elements in determining which dimensions form decision-space. Three scenarios are shown: control ($z_{\text{LHb}}=0.5$ in eq. (5)), lesioned LHb $(z_{\text{LHb}}=-5)$, and stimulated LHb $(z_{\text{LHb}}=5)$. The colors shown for each circuit element correspond to the decision-space that would be formed absent the influence of downstream circuit elements. **j-l**, Process by which action values are defined using decision-space. During a decision, a subset of decision-dimensions is selected, forming decision-space (**h**). This is represented mathematically through a diagonal matrix $S_{P}$ whose elements are set probabilistically to either 1 (dimension in decision-space) or 0 (dimension not in decision-space) (eq. (3)). Otherwise, mSPN activity is formulated similarly to sSPN activity. Rules corresponding to retained decision-dimensions are used to define action values $v_{j,P}$ for action j and pathway ***P*** (**k,l**). This is represented mathematically as multiplication of a vector $\beta_{j,P}$ by mSPN activity $s_{\text{mSPN},P}$, subtracted by a shift $\alpha_{j,P}$ and run through an activation function (eq. (4)). **m,n**, Action (or inaction) values $v_{j,P}$ for each action j and pathway P are used as drift rates (**m**) in a Merton process model (**n**). Discrete Merton processes obtained as a constant time step discretization of eq. (6) are run for each action simultaneously. At the time the first of the $v_{j,\text{direct}}$ processes reaches a defined threshold h, the corresponding action is enacted unless its inaction process $v_{j,\text{indirect}}$ has reached h first (eqs. (7),(8),(9)). Lines show the progress of processes towards a decision threshold for an example simulation. Histogram shows the decisions and deliberation times for the processes that reached the threshold first. **o,p,** Psychometric functions derived across multiple reward levels. In the modeled experiment, the subject is asked to evaluate the rewards and costs of two offers and either approach or avoid. The decision-space (**o**: 1D, **p**: 2D) affects choice patterns. **q,r,** Demonstration of a method by which decision-space can be inferred from sSPN activity, showing the utility of the mathematical formation of the model in connecting experimental inputs, sSPN activity, and choice. The method demonstrated here could be used to design an experiment in which the decision-space model is tested, or it could be used to explain differences in choice between groups, for instance control and disorder. In the simulation, two environmental inputs (e.g. temperature, music volume) across 100 sessions are classified based on decision-making phenotypes, for example based on reaction time, heart rate, eye movement (colors). Using the method visualized here, the decision-making phenotype classes are assigned one of four decision-space reference labels: 1) where decision-space is not formed, 2) where only the first decision-dimension is used to form decision-space, 3) where only the second decision-dimension is used to form decision-space, and 4) where both decision-dimensions are used to form decision-space. Synthetic data is generated by forming an arbitrary set of cortex→sSPN weights, using these to form sSPN activity, and then adding i.i.d. Gaussian noise. A linear regression is used to derive estimated decision-dimensions and assign hypothesized decision-spaces to each label. Choice is then examined with respect to the derived decision-dimensions. As expected, the regression slope (planes) corresponding to the 2D decision-space is roughly the sum of the regression slopes of the two 1D decision-spaces (**q**), and decisions correlate with the dimensions hypothesized to be used to form decision-space when choices are plotted against hypothesized dimensions (**r**). In the colormaps in **r**, action values are interpolated from an example set of observations (diamonds and Xs) via logistic regression, and a boundary line is drawn where the action value of “play music” equals the action value of “stand up.” (1) $s_{\text{sSPN},P}=\frac{1}{c_{P}}{\;W}_{P}{\;T}_{x_{P}}+b_{\text{sSPN}}$ (copied for convenience) (2) ${\text{daSNC}}_{i,P}=\frac{1}{1+\text{exp}\;\left(w_{\text{sSPN}\to \text{daSNC},i,P}\cdot {\;s}_{\text{sSPN},i,P}+\text{RMTg}-z_{\text{daSNC},i,P}\right)}$ (copied for convenience) (3) $s_{\text{mSPN},P}=\frac{1}{c_{P}}S_{P}{W_{P}}^{T}x_{P}$ (copied for convenience) (4) $v_{j,P}=\frac{1}{1+\text{exp}\;\left(-\beta_{j,P}{\;s}_{\text{mSPN},P}-\alpha_{j,P}\right)}$ (copied for convenience) (5) $\text{RMTg}=z_{\text{RMTg}}+z_{\text{LHb}}+z_{\text{GPi}}\cdot w_{\text{GPi}}\cdot \left[\begin{array}{c} {\text{s}}_{\text{sSPN},\text{direct}}\\ {\text{s}}_{\text{sSPN},\text{indirect}} \end{array}\right]$ (6) $dY_{j,P}=v_{j,P}\;dt+\sigma \;dW_{j,P},Y_{j,P}(t=0)=0$, where $W_{j,P}$ is a standard Wiener process (7) $t_{\text{action},j}=\underset{t}{\text{min}}\;\left\{t|Y_{j,\text{direct}}\geq h\right\}$ (8) $t_{\text{inaction},j}=\underset{t}{\text{min}}\;\left\{t|Y_{j,\text{indirect}}\geq h\right\}$ (9) $\text{action}=\text{arg}\;{\text{min}}_{j\in J}\;\left(Y_{j}\left(t_{\text{action,}j}\right)\right)$, where J is the subset of actions s.t. $t_{\text{action,}j}<t_{\text{inaction,}j}$

Our model describes how physiological interactions between elements of a striosome-centered circuit inform decision-making. (For extended reasoning behind our choice of circuit elements, see Supplementary Note 1.) **Striatum-projecting cortical neurons** that encode mixed information serve as the input to the modeled circuit. We assume for the purpose of our model that the information passed from the cortex to each striatal compartment is roughly similar (in actuality, there is much overlap with some differences, see Table 2 and Supplementary Note 2). The cortical neurons synapse on subpopulations of proximate SPNs which have been found to each encode distinct information^27^. During this process, fast spiking interneurons (**FSIs**) perform a normalization operation (Extended Data Fig. 1a). Mathematically, we represent this as a matrix $W_{P}$ dictating cortex-SPN connection weights for each pathway P (direct or indirect) mapping cortical activity $x_{P}$ to the coordinate space of sSPNs, where it undergoes divisive normalization by FSI activity $c_{P}$ and a shift in activity $b_{\text{sSPN}}$ to form sSPN activity $s_{\text{sSPN},P}$:

(1) $$s_{\text{sSPN},P}=\frac{1}{c_{P}}{W_{P}}^{T}x_{P}+b_{\text{sSPN}}$$

Information from sSPNs is then passed to dopaminergic neurons of the substantia nigra compacta (**daSNC**). daSNC neurons, like sSPNs, have been shown to be organized topologically into subpopulations that each encode distinct information^28^. Experimental evidence suggests that signals are passed from sSPN subpopulations to daSNC neurons in three ways (see Table 2): **A) dsSPN→daSNC**. Subpopulations of dsSPNs inhibit daSNC subpopulations directly via dendrite bouquets^26^. Thus, in our model, each dsSPN subpopulation inhibits a corresponding daSNC subpopulation. **B**) **isSPN→GPe→daSNC**. isSPNs send signals to daSNC neurons via GPe^16^, which inhibit daSNC subpopulations. Thus, in our model, each isSPN subpopulation disinhibits a corresponding daSNC subpopulation. **C) sSPN→GPi→LHb→RMTg→daSNC**. GPi integrates signals from many sSPN subpopulations through synapses that release both GABA and glutamate^29^, and the LHb, when activated by GPi, powerfully inhibits multiple of the dopaminergic subpopulations via RMTg^30,31^. So, in our model, shifts in this pathway lead to a shift across all daSNC subpopulations. Mathematically, we represent the three circuits as daSNC combining activity from sSPNs s_sSPN,*P*_ (with connection weights $w_{\text{sSPN}\to \text{daSNC},i,P}$ corresponding to each decision-dimension i and pathway P) with RMTg activity and an additive shift $z_{\text{daSNC},i,P}$:

(2) $${\text{daSNC}}_{i,P}=\frac{1}{1+\text{exp}\left(w_{\text{sSPN}\to \text{daSNC},i,P}\cdot s_{\text{sSPN},i,P}+\text{RMTg}-z_{\text{daSNC},i,P}\right)}$$

In addition to sSPNs, there are subpopulations of **mSPNs**, termed matrisomes^4^, that densely surround sSPN subpopulations. In our model, we hypothesize that these sSPN and mSPN subpopulations communicate with one another via dopamine release from the corresponding daSNC subpopulation (there are other sSPN→mSPN connections which we do not model that play more local roles, see Supplementary Note 3). There are multiple groups of these functionally connected sSPNs, daSNCs, and mSPNs. In the model, when a daSNC subpopulation is active, dopamine is released to the corresponding sSPN and mSPN subpopulations, resulting in enhanced or inhibited mSPN reception of cortical signal among the subpopulations, as shown in experimental work^32^. Mathematically, mSPN activity is defined similarly to sSPN activity, but for a diagonal matrix $S_{P}$ corresponding to the dopamine release that probabilistically defines the decision-space, with $P\left(S_{P,\text{ii}}=1\right)={\text{daSNC}}_{i,P}$:

(3) $$s_{\text{mSPN},P}=\frac{1}{c_{P}}S_{P}{W_{P}}^{T}x_{P}$$

mSPNs have been found to be primarily involved in motor functions, projecting to the GPi, SNr, and then to brainstem motor programs^33^. SPN activity (which is predominantly mSPN) has been shown to contribute to action selection and initiation^34^. The direct pathway has generally been implicated in promoting actions and the indirect pathway in preventing actions^35^. So, in our model, the output of the circuit is the definition of action values (the value of performing various actions, encoded by the direct pathway) and inaction values (the value of refraining from those actions, encoded by the indirect pathway) by mSPN signals on route to downstream regions. Mathematically, values $v_{j,P}$ (for each action/inaction j, pathway P) are defined based on mSPN activity, internal coefficients $\beta_{j,P}$, and priors $\alpha_{j,P}$ (which are set to arbitrary values that are constant across analyses, see **Common parameters,** Methods):

(4) $$v_{j,P}=\frac{1}{1+\text{exp}\left(-\beta_{j,P}{\;s}_{\text{mSPN},P}-\alpha_{j,P}\right)},$$

Based on these values, actions are either performed or refrained from over time. We model this using a Merton process model where the first process to hit a threshold is enacted (direct pathway) or refrained from (indirect pathway). See **Defining choice,** Methods.

**Fig. 1:**
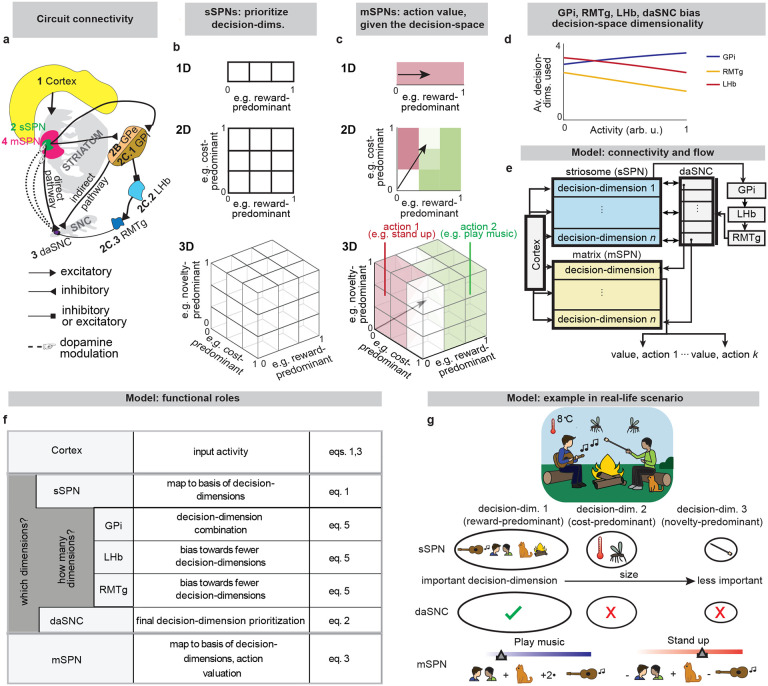
The circuit defines a decision-space for action valuation. **a**, The circuit of the cortex, striosomes (sSPN), globus pallidus externus (GPe), globus pallidus internus (GPi), lateral habenula (LHb), rostromedial tegmental nucleus (RMTg), dopaminergic neurons of the substantia nigra compacta (daSNC), and matrix neurons (mSPN). Numbers show the order of connection for the three subcircuits (1→2→3, 1→2→2B→3→4, 1→2C.1→2.C.2→2.C.3→3→4). **b**, During decision-making, sSPNs help to determine which decision-dimensions are most important. Important decision-dimensions are later formed into decision-space (grids of cubes or squares) when dopamine is released from daSNC to mSPNs. For example, a 1D decision-space might be constructed where decision-making is conducted using information predominantly about reward, or multi-dimensional decision-space might be constructed for situations where multiple decision-dimensions are important to the decision. **c**, After the decision-space is formed, mSPNs define action values (colors) based on cortical input (vectors) and the decision-space (grids). In a 1D decision-space, action values are assigned purely based on the activity of mSPN along a single decision-dimension. In multi-dimensional spaces, action values depend on the activity along multiple decision-dimensions. **d**, GPi, LHb, RMTg, and daSNC activities affect the number of decision-dimensions that will be used to form the decision-space. **e,f,** Circuit architecture (**e**) and functional role of circuit elements (**f**). The cortex encodes environmental and internal information. sSPN subpopulations each encode data along a decision-dimension. GPi→LHb→RMTg biases the circuit towards using more or fewer decision-dimensions. daSNC subpopulations, each corresponding to a decision-dimension, activate when their decision-dimension is important. This causes dopamine to be released to select mSPN subpopulations, and thus, a “decision-space” is formed from the basis of the important decision-dimensions. mSPNs define action values within the decision-space. **g**, Scenario where the cortex encodes signals about food, social, and environmental cues. A “reward-predominant” decision-dimension captures information about music, cat, fire, and social interaction; a “cost-predominant” decision-dimension captures information about temperature and mosquitoes; and a “novelty-predominant” decision-dimension captures information about marshmallows. The most important decision-dimensions (here, the reward-predominant decision-dimension only, assigned the checkmark) are retained. mSPN forms action values using rules corresponding to the retained decision-dimensions, that is, within the decision-space. Several actions (here, “play music” and “stand up”) are assigned values, and then decisions are made based on the values of possible actions.

Thus, our model is constructed based on the anatomy and physiology of the striosome-centered circuit. The physiological description also produces a simple and convenient geometric interpretation. If we let each SPN and daSNC subpopulation encode the principal components of cortical activity, such as could be learned via a modified Oja’s rule^36^, then the columns of $W_{P}$ become orthogonal. So, each SPN subpopulation can be thought of as encoding information along an axis of Euclidean space. We term these axes “decision-dimensions” and have evidence that they correspond to constructs such as reward, cost, or novelty (discussed in more detail below). We suggest that when dopamine is released to SPNs, selectively enhancing reception of cortical signal, decision-dimensions are effectively prioritized. Therefore, cortex, striosomes, and dopamine work together to form a “decision-space”, only focusing on decision-dimensions that are necessary based on context (Figs. 1b,c). In this light, sSPN and daSNC, via pairs of connected subpopulations that process information in parallel, serve the functional role of selecting *which* decision-dimensions should receive high priority. In particular, dsSPNs determine which decision-dimensions to use in the direct pathway, and isSPNs which decision-dimensions to use in the indirect pathway. On the other hand, GPi, LHb, and RMTg, by prioritizing or deprioritizing all daSNC subpopulations together, determine *how many* decision-dimensions should be prioritized (Fig. 1d, Extended Data Figs. 1b–i).

Importantly, the advantage of this formation is not only conceptual, but practical for linking physiology to decision-making (Figs. 1e–g, Extended Data Figs. 1j–p). Distinct sSPN and/or daSNC subpopulations have been found to encode, for instance, reward^12,14,15,28^, cost^12,14,15,28^, or novelty^37^. Thus, we might imagine that decision-dimensions could correspond loosely to reward level, cost level, or novelty level. If this is the case, a logical prediction of the model arises: we would expect a low-dimensional decision-space to be formed during a simple choice (e.g. between two rewards) and a high-dimensional decision-space to be formed during a more difficult choice (e.g. between offers which each have benefits and costs that must be weighed in order to solve the problem). This hypothesis, if proven, would allow us to infer the decision-spaces of behaving rodents or humans simply by regressing sSPN activity on experimental parameters (for example, temperature or music volume), as we demonstrate using synthetic data (Extended Data Figs. 1q,r). For example, a significant correlation between sSPN activity and novelty level would indicate the existence of decision-dimension that corresponds roughly to novelty (**Inferring decision-space from SPN activity and choice,** Methods). We sought to determine if this hypothesis is supported by experimental physiological data collected during decision-making.

### Support for the model: context-dependent sSPN physiology matches model predictions.

**Extended Data Fig. 2:**
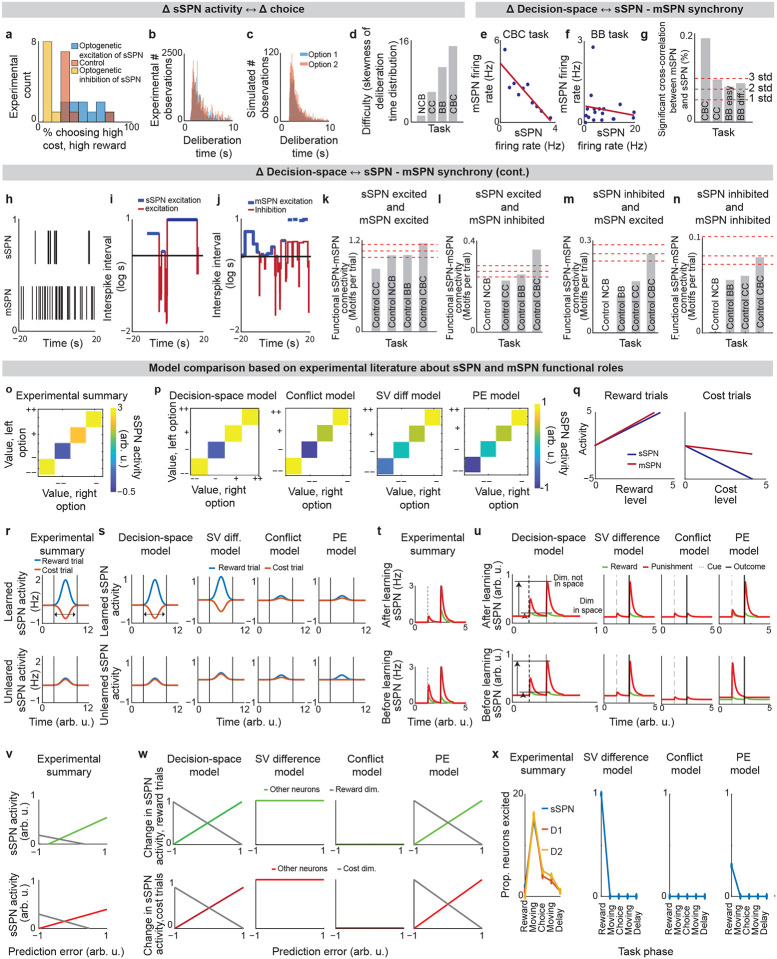
**Decision-space model validation and comparison to alternative models, Related to** Fig. 2. **a**, Summary of experimental results of optogenetic manipulation during a conflict task in Friedman et al. (2015). Resembles the model in Fig. 2b. **b,c,** Experimental deliberation time distribution (6 animals, 35 sessions) (**b**), which is successfully modeled using the Merton process model (**c**). Distribution is for the benefit-benefit task (concentration 70%). Experimental data here and throughout the figure is analyzed from the Corticostriosomal Circuit Stress Experiment database. **d**, Skewness of the deliberation time distribution, which is used to estimate task difficulty. The CBC task had a deliberation time distribution that was more skewed than the non-conflict tasks. Tasks: NCB = non-conflict cost-benefit (4 rats, 27 sessions, 1250 trials), CC = cost-cost (7 rats, 25 sessions, 1852 trials), BB = benefit-benefit (7 rats, 128 sessions, 7762 trials), CBC = cost-benefit conflict (8 rats, 69 sessions, 3921 trials). **e,f,** Analysis of relationship between sSPNs and mSPNs during decision-making. sSPN and mSPN neurons are significantly more correlated in tasks that require integration of reward and cost versus only reward or only cost. e shows representative examples from the cost-benefit conflict task (CBC, both reward and cost) and **f** shows the benefit-benefit task (BB, only reward), respectively. **g**, The CBC task had significantly more correlated (Pearson’s r^2^ > 0.4 and significance < 0.05) pairs than the tasks which required integration of only reward or only cost. Confidence intervals (dashed red lines, 1,2,3 standard deviations) are estimated based on shuffled data. NCB = non-conflict cost-benefit (sSPNs = 14, mSPNs = 260), CC = cost-cost (sSPNs =46, mSPNs = 400), CBC = cost-benefit conflict (sSPNs = 84, mSPNs =717), BB = benefit-benefit easy (sSPNs = 50, mSPNs =515, chocolate milk concentration <50), BB = benefit-benefit difficult (sSPNs =33, mSPNs = 731, chocolate milk concentration >=50) **h-j**, Process by which we identify functionally connected sSPN and mSPN neurons. Spiking times (**h**) are used to find inter-spike intervals (**i**,**j**) for sSPN (top rows) and mSPN (bottom rows) that were recorded simultaneously during decision-making. Intervals above the median interval (black line) are classified as inhibition (blue) and below median are classified as excitation (red). Functional connections are determined based on whether excitation or inhibition of sSPN followed by excitation or inhibition of mSPN. **k-n,** Significantly more sSPN and mSPN neurons were functionally connected during decisions that required integration of reward and cost (CBC task) than the other tasks for all types of connections: sSPN excited and mSPN excited (**k**), sSPN excited and mSPN inhibited (**l**), sSPN inhibited and mSPN excited (**m**), and sSPN inhibited and mSPN inhibited (**n**). Tasks: NCB = non-conflict cost-benefit (sSPNs = 14, mSPNs = 260), CC = cost-cost (sSPNs =46, mSPNs = 400), BB = benefit-benefit (sSPNs =83, mSPNs = 1246), CBC = cost-benefit conflict (sSPNs = 84, mSPNs =717) **o**, Mean sSPN activity should track decision-space dimensionality. Tasks in Friedman et al. (2015), plotted in order of difference between reward and cost, are assessed for likely decision-space dimensionality based on task difficulty (Extended Data Fig. 2d). Those with higher task difficulty are assigned lower sSPN activity per the model in Fig. 2c. **p**, Three alternative models applied to the task in Friedman et al. (2015). The subjective value (SV) model assumes that sSPN encoded the relative action values of the two arms of the T-maze. The conflict model assumes that sSPN activity is inversely proportional to the amount of conflict in the task. The prediction error model compares expected value entering the task with reward or cost obtained on the maze. The conflict method resembles experimental results but not the SV difference model or the conflict model. **q,r,** Summary of differences in sSPN activity across trials in the operant conditioning task in Friedman et al. (2020). mSPNs and sSPNs were active during reward trials, suggesting per the model in Fig. 2c that the decision-space was not formed (**q**). sSPNs were less active during the cost trials and were differently active than mSPNs, suggesting formation of a low-dimensional decision-space per the models in Figs. 2c,d (**r**). **s**, Prediction of sSPN activity for the task in **r** for the decision-space model and three alternative models. sSPN activity scales with overall subjective value in the task, so the subjective value model successfully interprets the experimental results. **t,u,** Experimental results are summarized from Xiao et al. (2021) (**t**). Prediction of the decision-space model and three alternative models of sSPN subpopulation activity at the cue and during the outcome period of a Pavlovian conditioning task (**u**). Per the decision-space model, sSPN subpopulations should respond similarly to the cue and to the outcome because associated data is likely mapped along the same decision-dimension. The subjective value model expects more activity during the outcome period when reward is administered. The prediction error model expects more activity at the cue after learning. There was no conflict in the experimental setup. **v,w,** Experimental results are summarized from Bloem et al. (2022) (**v**). Predictions of the decision-space model and the three alternative models of sSPN activity during a probabilistic Bandit task (**w**). The decision-space model expects that sSPN activity will reveal prediction errors, as does the prediction error model. The subjective value model instead anticipates that activity will track overall value regardless of prediction error. There was no conflict anticipated by conflict model. **x**, In the value-guided choice task in Weglage et al. (2021), activities of all neuron types recorded (sSPN, dmSPN, imSPN) resembled one another over phases of the task and did not solely track subjective value, prediction error, or conflict. As shown in Fig. 2d, the experimental finding is an expectation of the decision-space model when a high-dimensional decision-space is formed.

**Fig. 2:**
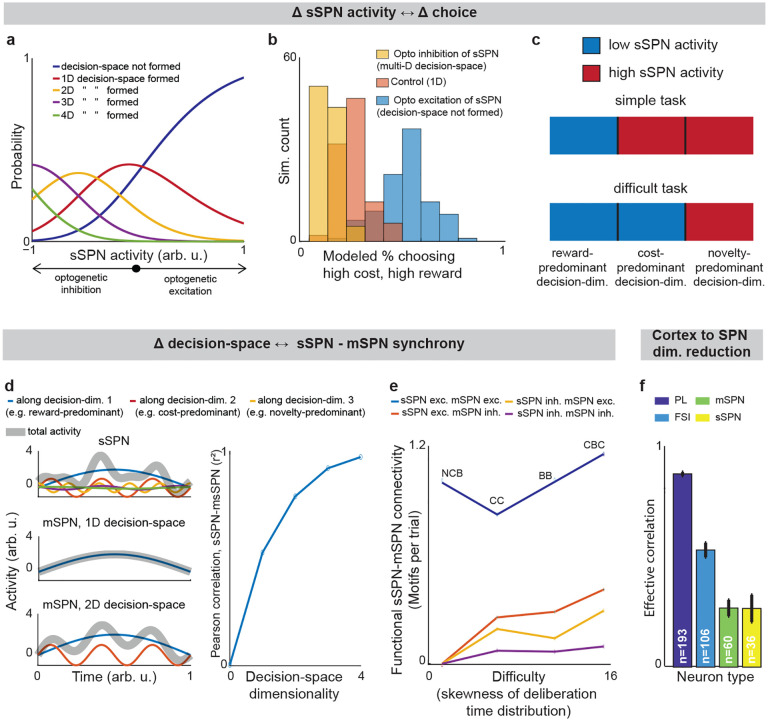
Evidence of decision-space formation in sSPNs and mSPNs. **a**, Changes to sSPN activity, for instance during optogenetic manipulation, cause changes in the modeled number of decision-dimensions used to form decision-space. In the analysis, $b_{\text{sSPN}}$ in eq. (1) is incremented from −1 (low activity) to 1 (high activity). **b**, More consistent choices are made at lower sSPN activity. 100 choices are simulated for each of the three decision-space scenarios. Modeled choices are made between a high-cost, high-reward option and a low-cost, low-reward option. The multi-D decision-space applies rules for both reward and cost, the 1D decision-space rules about only reward, and the “decision-space not formed” case applies neither. Then action values and choice are derived. **c**, An sSPN subpopulation has reduced activity when it is used to form decision-space. Therefore, mean sSPN activity is different in simple tasks requiring low-dimensional decision-space versus difficult tasks requiring high-dimensional decision-space. **d**, When a one-dimensional decision-space is formed, sSPN (top-left panel) and mSPN (middle-left) activities have low correlation over time. As more decision-dimensions are used to form the decision-space, sSPN and mSPN activities increasingly correlate (bottom-left). Pearson’s correlation between sSPN and mSPN activities (right panel). **e**, Experimental data showing that task difficulty (measured through skewness of the deliberation time distribution) increases with functional sSPN-mSPN connectivity (measured through Granger causality) during a decision. Tasks: NCB = non-conflict cost-benefit (sSPNs = 14, mSPNs = 260), CC = cost-cost (sSPNs =46, mSPNs = 400), BB = benefit-benefit (sSPNs =83, mSPNs = 1246), CBC = cost-benefit conflict (sSPNs = 84, mSPNs =717). The CBC task has significantly more motif counts per trial (p<0.003, compared to shuffled data). **f**, Experimental data showing that simultaneously recorded SPNs have less correlation (measured as effective correlation) than FSIs or prelimbic cortical neurons. This indicates dimensionality reduction from cortical neurons to SPNs. Significances of difference from cortical neurons: FSI p<10^−18^, mSPN p<10^−45^, sSPN p<10^−63^.

We began by asking whether the results of the physiological sSPN literature support a link between the decision-space (which our model postulates is driven by sSPN activity) and task difficulty (inferred from experimental inputs, for instance a simple task with reward only versus a difficult task with conflicting rewards and costs). We began with the experimental literature on sSPNs. In one experiment^11^, sSPNs were optogenetically stimulated (or inhibited) during a rodent conflict decision-making task. Per our model, this should cause inhibition (or disinhibition) of daSNC neurons, leading to reduced (or enhanced) dopamine release to SPNs, producing a lower- or (higher-) dimensional decision-space. Indeed, the stimulation led to choices indicative of decision-making using few informational dimensions, while inhibition led to choices indicative of decision-making using multiple informational dimensions (Figs. 2a,b, Extended Data Fig. 2a, **Effect of sSPN activity on decision-space** and **Effect of decision-space on choice,** Methods). A second experiment^11^ tested, reversely, the activity of striosomes during simple versus difficult tasks. As the model expects, striosomal activity during simple tasks resembled the levels observed during optogenetic excitation, whereas striosomal activity during difficult tasks resembled the levels observed during optogenetic inhibition (Fig. 2c, Extended Data Figs. 2b–d). In another study, striosome activity was lower among rodents that learned a difficult reversal learning task than among rodents that did not^10^. Thus, there appears to be a relationship between sSPN physiology and task difficulty in the direction expected by our model.

### Support for the model: context-dependent sSPN-mSPN synchrony matches model predictions.

Importantly, the result above does not distinguish our model from the alternative, simpler explanation that sSPNs might collectively encode task difficulty. We could term such a model a “conflict model,” where sSPN activity tracks the overall conflict present in a task (see Table 3). To determine whether it was changes to sSPN subpopulations driving the overall change in sSPN activity (i.e. decision-space), rather than a general effect, we analyzed the paired activities of sSPNs during simple versus difficult tasks using the Corticostriosomal Circuit Stress Experimental database^3^. We hypothesized that a greater number of sSPNs and mSPNs would be functionally connected during difficult tasks. This prediction arises from the circuit connectivity in our model, where sSPNs are functionally connected to mSPNs via daSNC, and each additional prioritized subpopulation causes more sSPN→daSNC→mSPN modulation (Fig. 2d). This could be observed, for instance, as an increase in striosome and matrix correlation as decision-space increases. This hypothesis is also inspired by the observation that striosome and matrix activity has been found to roughly track one another over time in a difficult task^13^.

To test this, we analyzed the synchrony between striosome neurons and matrix neurons during simple decisions (that forced choice between either two rewards or two costs) and during difficult decisions (that forced choice between offers that contained both rewards and costs together). We measured synchrony as the cross-correlation between sSPN activity and mSPN activity over the period of the task. To control for possible physiological differences across the phases of the rats’ movements, we also developed a custom Granger causality-based tool. Per both metrics, synchrony was significantly higher in the difficult task. Further, synchrony scaled with the difficulty with which the rats treated the task, as measured based on deliberation time (Fig. 2e, Extended Data Figs. 2e–n**, Connected SPNs through cross-correlation** and **Connected SPNs through Granger causality,** Methods). Thus, rather than sSPNs encoding conflict in their general level of activity, there appears to be an important relationship between sSPN and mSPN subpopulations during high-conflict tasks. Our analysis does not confirm that sSPNs have a causal effect on mSPNs. However, if this is assumed, the evidence suggests enhanced modulation of mSPN subpopulations by sSPN subpopulations during the difficult tasks, that is, a formation of a higher-dimensional decision-space.

### Support for the model: Dimensionality reduction from the cortex to SPNs.

Notably, the synchrony analysis above demonstrates the use of subpopulations during context-dependent decision-making, but it does not test whether those subpopulations correspond to decision-dimensions. There is, however, evidence that SPNs encode what we term decision-dimensions. Experimental work has demonstrated that distinct SPN subpopulations encode different information, and that these subpopulations persist across days^27^. Further, our analysis suggests that dimensionality reduction occurs from the cortex to SPNs, as it does in our model during the mapping of information to a basis of decision-dimensions. Cortical neurons had the most coordinated activities over time (measured as effective correlation^38^), then FSIs, followed by sSPNs and mSPNs (Fig. 2f, **Analyzing neural dimensionality reduction,** Methods). This would suggest a higher-dimensional representation in cortex, where neurons encode similar information over time, than in the downstream regions, where information is compactly encoded in neurons that behave differently over time. Thus, it seems that cortex→SPN dimensionality reduction occurred during the tasks, like in our model the mapping from high-dimensional cortical information to a basis of SPN decision-dimensions.

### Support for the model: Strong alignment to the experimental literature on SPNs compared to alternative models.

The analysis to this point is based on a selection of the experimental literature relating sSPN activity to mSPN activity and choice. We wished to test the model more broadly using a range of experimental results. To this end, we devised five tests that link sSPN activity to choice, each verifiable with the experimental literature, that might support or reject the decision-space model (Extended Data Figs. 2o–x, Table 3). As benchmarks, we also constructed, from roles commonly assigned to sSPNs, four alternative models in which sSPNs encode 1) conflict, 2) subjective value, 3) prediction error, or 4) actions. We found that while the alternative models each can be used to interpret a subset of the experimental evidence, only the decision-space model aligned to the breadth of it (Table 4). A selection of experimental studies on GPi, LHb, and daSNC also align with the decision-space model (see Tables 5–8), thus offering a new lens through which to interpret their functions.

### Inability to form a high-dimensional decision-space in disorders.

**Fig. 3:**
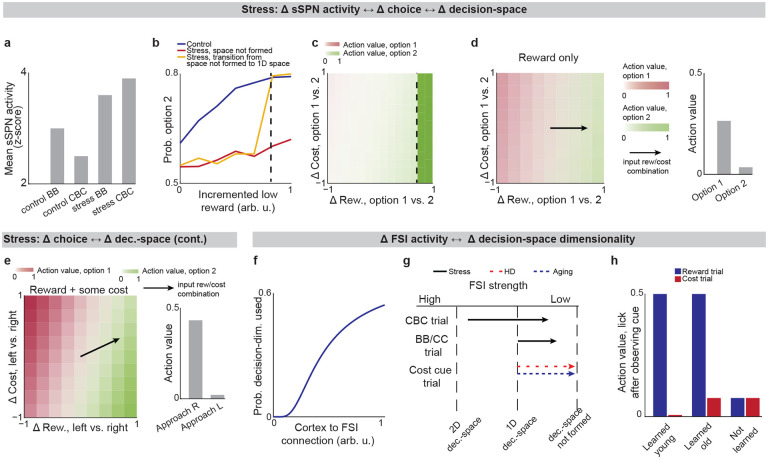
Lower-dimensional decision-spaces are produced in stress, aging, and HD, affecting decisions. Data was taken from experiments where rodents performed the cost-benefit conflict (CBC) task, in which rodents had to select between a high cost-high reward option and a lower cost-lower reward option. Two behavioral control tasks were also used: the benefit-benefit (BB) task, in which rodents selected between a high reward option and a low reward option (equal and minimal cost for both), and the cost-cost (CC) task, in which rodents selected between a high cost option and a low cost option (equal and minimal reward for both). **a**, Summary of the experimental finding that sSPN activity is increased after stress, especially during the more difficult CBC task. **b,c,** Modeled psychometric functions (**b**) and action values across experimental conditions (**c**) for a rodent in a T-maze task after chronic stress. The circuit forms a 1D decision-space only after reward exceeds a critical concentration (dashed line). At this point, the stress-group rodents switch from choosing the options roughly evenly to most often turning right towards the lower-cost option. Psychometric functions resemble experimental decision-making data. **d,e,** After stress, a low-dimensional decision-space may be used by default (**d**) but a higher-dimensional decision-space may sometimes form during difficult tasks, for example those with both reward and cost (**e**). This leads to the counterintuitive result that adding cost to an offer (vectors on colormaps) can increase its action value (bar plots). **f**, Cortex→FSI connection strength affects decision-space dimensionality by altering the propensity of a decision-dimension to form decision-space. **g**, Cortex→FSI connection strength is reduced in stress, Huntington’s disease, and aging. This leads to lower-dimensional decision-space. **h**, Model of an operant conditioning task in Friedman et al (2020). Modeled action values for licking versus not licking in an operant conditioning task. There are two tasks: 1) responding to a reward cue by forming decision-space from a reward-predominant decision-dimension, and 2) likewise for cost. “Learned young” succeeds at 1 and 2, “learned old” at 1 but not 2, and “Huntington’s disease” at neither. Resembles experimental licking rates.

We next applied our model to the experimental literature on neuropsychiatric disorders, wondering if it could offer insight into disorders associated with sSPN changes. Interestingly, in an experimental study on chronic stress^3^, sSPNs were hyperactive compared to controls during the most difficult task but less affected during the simpler tasks (Fig. 3a). Meanwhile, the rodents were less adherent to reward level only in the difficult task, suggesting dysfunction in processing reward and cost together (Figs. 3b,c**, Effect of decision-space on choice,** Methods). Thus, we wondered if post-stress sSPN hyperactivity could cause altered choices due to a reduction in the dimensionality of the decision-space, similar to the model in Fig. 2b where optogenetic excitation reduced the decision-space in controls.

**Extended Data Fig. 3:**
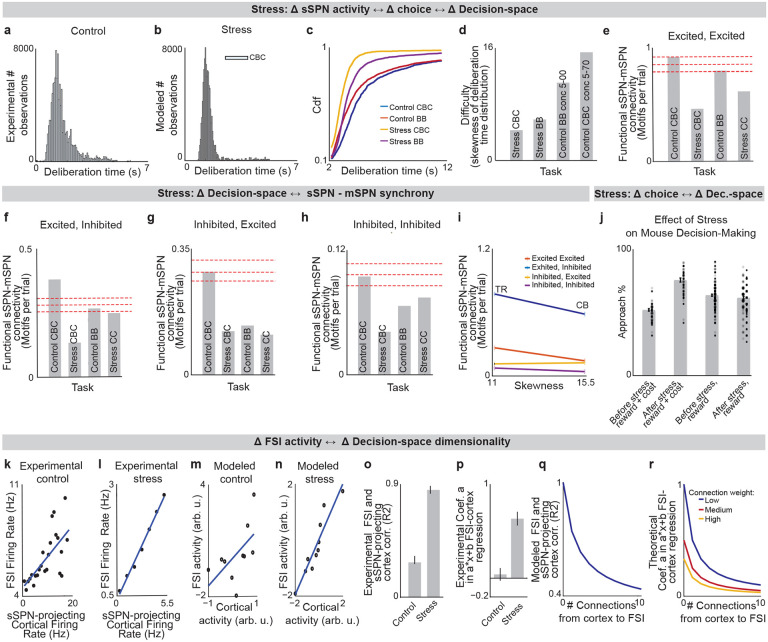
Analysis of neural and decision-making data shows that decision-space is changed after stress, Related to Fig. 3. (84/85) **a,b,** Deliberation time distributions of rodents performing the cost-benefit conflict task before stress (**a**) are less skewed and have shorter deliberation time than after stress (**b**, control: 8 rats, 198 sessions, 11683 cells; stress: 5 rats, 138 sessions). **c,d,** Cumulative distribution functions (**c**) and distribution skewness (**d**) of deliberation time show that after chronic stress, the task involving integration of both reward and cost (CBC task) changes from producing the slowest (blue) to the quickest (yellow) decisions. CBC choice was slowest in control rats (p < 0.0001, KS-test). After stress, CBC choice was faster than in the other tasks (p<0.0001). Control CBC task: 69 sessions, 8 rodents; Control BB task: 128 sessions, 7 rodents; Stress CBC task: 34 sessions, 5 rodents; Stress BB task: 104 sessions, 5 rodents. **e-h,** sSPNs and mSPNs were more functionally connected during a difficult (CBC) task before stress than after, per all possible types of connection: sSPN excited, mSPN excited (**e**), sSPN excited, mSPN inhibited (**f**), sSPN inhibited, mSPN excited (**g**), and sSPN inhibited, mSPN inhibited (**h**). Significance levels, depicted by dashed lines, show one (bottom), two (middle), and three (top) STD for functional connections calculated from shuffled control data. Control cost-benefit conflict (CBC) task: 92 pairs, 8 rodents; Tasks: Control BB = benefit-benefit (sSPNs =83, mSPNs = 1246), Control CBC = cost-benefit conflict (sSPNs = 84, mSPNs =717), Stress CBC = cost-benefit conflict (sSPNs =41, mSPNs =898), Stress BB = benefit-benefit (sSPNs = 156, mSPNs = 2813). **i**, Deliberation time distribution skewness is no longer linked to functional connectivity after stress. **j**, Experimental data that inspires the model in Fig. 3h. Mice that underwent chronic stress approached the lower-reward arm of the T-maze less when a small cost was added (“reward + cost” = cost-benefit conflict task, “reward” = benefit-benefit task). Dots = individual sessions, bar = mean across trials. There is significant difference (p<10^−19^, paired t-test) in choice for the CBC task before and after stress, and nonsignificant difference in choice for the BB task before and after stress (p=0.10). CBC, before stress: 17 rodents, 38 sessions; CBC, after stress: 13 rodents, 34 sessions; BB, before stress: 23 rodents, 114 sessions; BB, after stress: 14 rodents, 116 sessions. Our model interprets this result as due to differences in the decision-space. **k-n,** Representative examples of simultaneously recorded FSIs and prelimbic cortex neurons firing rates before (**k**, Pearson’s R=0.46) and after (**I**, R=0.99) chronic stress. After stress, there is less coordination between the connected pairs. This can be modeled as a reduction in the number of cortical neurons that synapse to each FSI (**m,n**). **o-r**, In general, the neuron pairs in rats that underwent chronic stress had significantly higher correlation (**o**, p<10^−18^) and significantly higher values of slope “a” in their a·x+b linear regression fits (**p**, p<10^−6^). Control: 7 rodents, 78 neuron pairs; Stress: 4 rodents, 37 neuron pairs. This suggests, per our modeling, that there are fewer connections from cortical neurons to FSI after stress. Modeled squared Pearson correlation coefficient (**q**) and slope “a” parameter in the a·x+b fits (**r**) are shown when the connection between cortical and FSI neurons are altered in two ways: 1) through a reduction in the number of connections, and 2) through a reduction in the strength of each connection (i.e. connection weight). This experimental evidence is aligned with a reduction in the number of connections, suggesting that FSI normalization is disrupted after stress, leading to higher sSPN activity and thus formation of lower-dimensional decision-spaces.

Our analysis supports this. Animals made decisions in the cost-benefit conflict task more quickly after stress, as if the task were less difficult (Extended Data Figs. 3a–d**, Defining decision difficulty by task,** Methods). Meanwhile, after stress, choices involving both reward and cost no longer had more functionally connected sSPNs and mSPNs than the simple tasks, suggesting a change to the decision-space as well as a general shift in activity. In fact, synchrony was similar across tasks and to the simple tasks for controls (Extended Data Figs. 3e–i). Thus, after stress, the rodents both showed both neural signatures aligned with a low-dimensional decision-space and choices aligned with processing of information in a low-dimensional way.

The inability to form a high-dimensional decision-space can also explain a counterintuitive finding that stress causes rodents to prefer a reward-cost combination over a reward presented without cost (Extended Data Fig. 3j**, Changes to choice after adding cost to a reward offer,** Methods). In classic economic theory, the addition of cost to a reward typically makes a good less attractive^39^ and thus our observation cannot be readily explained. In contrast, the decision-space model offers a simple explanation: cost can increase offer attractiveness in instances where cost level causes a transition from a default low-dimensional decision-space to a higher-dimensional decision-space, as we hypothesized is the case after stress in Figs. 3b,c. In these cases, the rules encoded by mSPNs can assign a higher value to accepting versus avoiding an offer when reward and cost are considered rather than only reward (Figs. 3d,e). In other cases, decisions are predicted to resemble those predicted by classic theory, for example in cases where either a one-dimensional (as in Fig. 3d) or two-dimensional (Fig. 3e) decision-space is used across cost levels.

**Extended Data Fig. 4:**
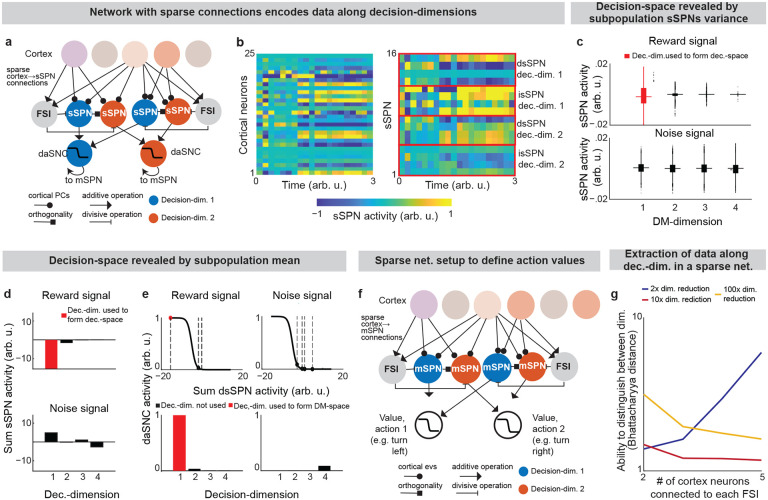
Mean and variance of SPN activities reveals decision-space. **a,b**, In this figure, we demonstrate that SPNs can encode activity along decision-dimensions successfully even in large, sparse networks, as exist in the brain, by considering an instance of the model where there are sparse connections between cortical neurons (n=50), FSIs (n=10,000), and SPNs (40,000 sSPNs, 40,000 mSPNs). sSPNs each encode activity along a principal component of a randomly sampled set of cortical neurons C (**a**). So, when activity changes in C (for example, during the reward cue in **b**, left panel), sSPNs (**b**, right panel) that each encode data along an ith principal component respond somewhat similarly to one another. See ***Instance 2: sparse connectivity and feedforward,*** Methods. **c**, Decision-space is revealed from the variance of sSPN activities, aligning to the experimental result in Fig. 2e. Modeled sSPN subpopulations have greater variance when there is high-magnitude cortical signal along a corresponding decision-dimension (e.g., an sSPN subpopulation corresponding to a reward-predominant decision-dimension in response to a reward cue). Summaries are shown of the activities of 10,000 dsSPN (top row). Two types of cortical signal are passed to SPNs: one with reward (left column, matches the example used in Fig. 4) and one with Gaussian white noise (right column). To produce the network, 10,000 groups of 4 randomly sampled cortical neurons (notated as the set C) are connected to an FSI and 4 dsSPNs (or isSPNs) and 4 dmSPNs (or imSPNs) for each pathway. The activity on a given sSPN which receives projection from $C,{\text{sSPN}}_{s,C}$, is defined mathematically based on the weights from cortical neuron q to $\text{sSPN}s,w_{q\to s}$, the activity of the FSI which received projection from $C,{\text{FSI}}_{C}$, and an additive shift that represents the relative activity of all sSPN neurons, $b_{\text{sSPN}}$ (eq. (10)). **d**, Decision-space is revealed by the mean of sSPN activities, aligned with the model in Fig. 2c. An sSPN subpopulation has lower mean activity when there is high-magnitude cortical signal along a corresponding decision-dimension. **e**, Selection process by which daSNC neurons determine which decision-dimensions to include and to not include in decision-space, illustrating the construction of decision-space from sSPN activity. Lines are daSNC activation functions. Placement along the x-axis (emphasized by dashed lines) is the subpopulation average activity in **d**. Mathematically, daSNC neuron corresponding to decision-dimension i and pathway $P,{\text{daSNC}}_{i,P}$, is computed as the weighted average of sSPNs corresponding to that decision-dimension and pathway, shifted by RMTg activity RMTg and a daSNC biasing factor $z_{\text{daSNC,}i,P}$, all passed through an activation function (eq. (11)). **f**, Correlate to the sSPN-centered subnetwork described in **a** for mSPN, illustrating how action values could be defined by a network with sparse connections. The activity of a given mSPN which receives projection from $C,{\text{mSPN}}_{m,C}$, is defined similarly to an sSPN but for term representing dopamine signaling from the daSNC neuron corresponding to decision-dimension i and pathway P to an mSPN corresponding to the same decision-dimension and pathway, $d_{i,P}$ (eq. (12)). **g**, Bhattacharya distance between the distributions of SPNs encoding data along the reward-predominant and cost-predominant decision-dimensions. sSPN can correctly differentiate reward from cost signal despite sparse cortico-striatal connectivity. Lines show the averages of 1000 simulations. This result demonstrates the feasibility of data along decision-dimensions being encoded by neural populations with sparse connections. (10) ${\text{sSPN}}_{s,C}=\frac{1}{\left|C\right|}\sum_{q\in C}\;\frac{w_{q\to s}{\text{cortex}}_{q}}{{\text{FSI}}_{C}}+b_{\text{sSPN}}$ (11) ${\text{daSNC}}_{i,P}=\frac{1}{1+\text{exp}\;\left(\frac{1}{n_{\text{sSPN}}}\sum_{s\in i,P}\;\;w_{s\to \text{daSNC},i,P}\cdot {\text{sSPN}}_{s}+\text{RMTg}-z_{\text{daSNC},i,P}\right)}$ (12) ${\text{mSPN}}_{m,C}=\frac{d_{i,P}}{\left|C\right|}\sum_{q\in C}\;\frac{w_{q\to m}{\text{cortex}}_{q}}{{\text{FSI}}_{C}}$

Cortex→FSI connectivity is impacted by chronic stress^3^ (Extended Data Figs. 3k–r, **Analyzed cortex-FSI connectivity and Modeled cortex-FSI connectivity after stress,** Methods), leading to hyperactive sSPNs. Our model suggests that this causes the formation of a lower-dimensional decision-space (Figs. 3f,g), which would lead to lower variance and higher mean of SPN subpopulations (Extended Data Fig. 4). Notably, the cortex→FSI connection is also impacted in Huntington’s disease and aged rodents^10^, raising the possibility that an inability to form a high-dimensional decision-space during difficult decisions is a feature of multiple health conditions. Supporting this hypothesis, a model where Huntington’s disease and aged subjects use a lower-dimensional decision-space produces action values that follow the trend of experimental choice (Fig. 3h, **Effect of decision-space on choice,** Methods).

**Extended Data Fig. 5:**
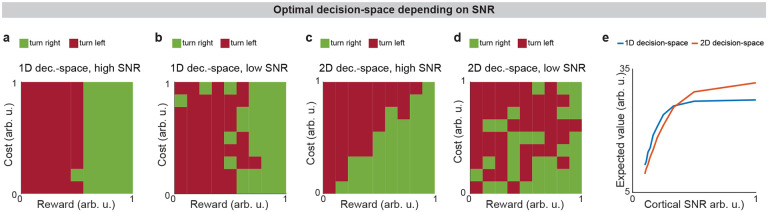
Decision-space is differentially constructed based on cortical signal-to-noise ratio (SNR). **a-d,** Modeled T-maze task where an animal turns right to choose a reward/cost offer or turns left to avoid it. Cases where there is high cortical SNR (**a,c**) or low cortical SNR (**b,d**) and a 1D decision-space (**a,b**) or 2D decision-space (**c,d**) are formed. Modeled “turn right” actions are considered successful (positive value) when reward > cost, and “turn left” when reward < cost. 2D decision-spaces lead to more value when there is low cortical SNR (**c**) but not when there is high SNR (**d**). **e**. Choices using different types of decision-spaces have different expected reward minus cost (expected value), depending on cortical SNR.

Interestingly, our model expects low dimensional decision-space to be beneficial in disorder conditions. A feature of chronic stress^3^ and schizophrenia^40^ is reduced cortical signal-to-noise ratio (SNR) and disrupted cortical signaling. In these conditions, a low dimensional decision-space is theoretically optimal because only the highest-priority decision-dimensions carry enough signal to outweigh the drawback of noise (Extended Data Fig. 5**, Effect of cortical SNR on choice,** Methods). Our analysis raises the possibility that FSIs help steer the circuit towards a helpful decision-space in disorders.

### Functional roles of dsSPNs, isSPNs, dmSPNs, and imSPNs.

sSPNs and mSPNs have been found to be distributed between the direct and indirect pathways of the striatum^24,41,42^, with dopamine release differently affecting sSPNs versus mSPNs and also dSPNs versus iSPNs^20,32,43^. Various neuropsychiatric disorders are associated with disturbed sSPN versus mSPN^2^ and direct versus indirect pathway balances^44^. Thus, the compartments likely play distinct functional roles in decision-making, including in disorders.

**Fig. 4:**
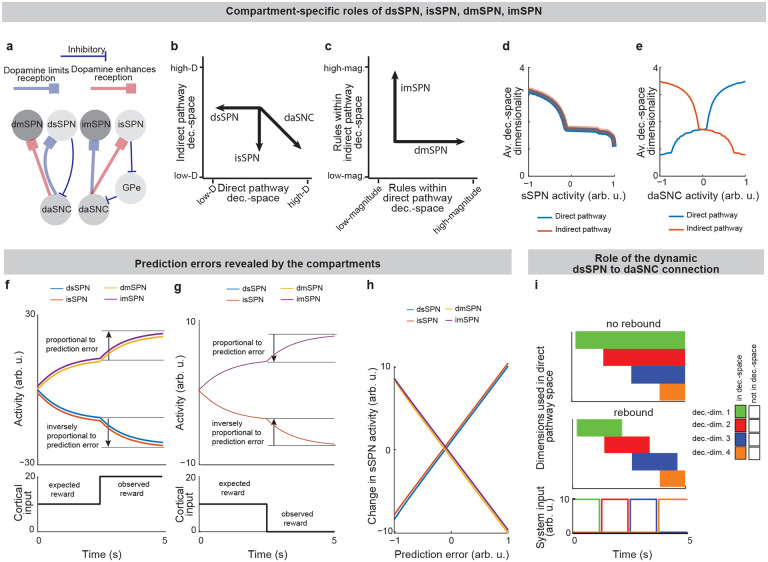
dsSPNs, isSPNs, dmSPNs, and imSPNs serve unique roles in constructing and using the decision-space. **a**, dsSPNs inhibit daSNC subpopulations and isSPNs disinhibit daSNC subpopulations via their connection through GPe. Dopamine lengthens upstates in dmSPN, enhancing reception of cortical signal, and shortens upstates in imSPN, limiting reception of cortical signal. For connectivity details, see Table 2. **b**, As a result of the circuit connectivity, two decision-spaces are constructed in parallel, one in the direct pathway and one in the indirect pathway. Circuit elements, when active, bias the dimensionality of the direct pathway decision-space, the indirect pathway decision-space, or both (vectors). **c**, Once the decision-spaces are formed, dmSPN activity amplifies the information along the direct-pathway decision-dimensions, and imSPN activity amplifies the information along the indirect-pathway decision-dimensions. **d**, Simulations where dsSPN or isSPN activity is incremented and the dimensionalities of the decision-spaces are counted at each time step. When overall sSPN activity changes, the dimensionalities of the direct and indirect decision-spaces change in the same direction. For example, at low sSPN activity, actions might be performed or refrained from based on careful consideration of many decision-dimensions, while at high sSPN activity, actions might be performed based on only reward or refrained from based on only cost. **e**, Similar, but overall daSNC activity is incremented. When daSNC activity changes, the dimensionalities of the direct and indirect decision-spaces change in opposite directions. For example, at high daSNC activity, actions might be performed based on careful consideration of many decision-dimensions or refrained from based on only cost, potentially leading to impulsivity. Meanwhile, at low daSNC activity, actions might be performed based on only reward or refrained from based on careful consideration of many decision-dimensions, potentially leading to low motivation. **f,g**, SPN signaling of positive (**f**) or negative (**g**) prediction error. Cortical input from one source, for instance a bell predicting a reward, begins at 0s. At 2.5s, reward information from a different source, for instance the administration of chocolate milk, is higher (**f**) or lower (**g**) than predicted by the first source, leading to a prediction error. In this example, the dsSPN, isSPN, dmSPN, and imSPN subpopulations corresponding to reward-related information reveal the prediction error, and not other SPNs. **h**, The simulations in **g** and **h** are run for incremented differences in expected versus observed reward, from −1 (negative prediction error) to +1 (positive prediction error), demonstrating that each population changes its activity roughly proportionally to prediction error. **i**, 1.25s pulses of input from the cortex (bottom panel) are applied along the four decision-dimensions in succession. A dynamic connection weight from dsSPN to daSNC facilitates the rapid de-prioritization of no longer required decision-dimensions in the “rebound” scenario, in which a period of dsSPN inhibition is followed by a rebound in daSNC activity above baseline levels (middle panel). This effect is removed in the “no rebound” scenario (top panel). In the model, the longer sSPN inhibits daSNC, the more the connection weight decreases in strength, leading to daSNC receiving decreasing signal about the importance of the decision-dimension. Then, when sSPN ultimately signals that the decision-dimension is no longer important, this signal is enhanced upon reception by daSNC.

Because dsSPNs connect to dmSPNs through daSNC in our model, and isSPNs to imSPNs through GPe and daSNC, two decision-spaces are formed in parallel, one related to each pathway (Fig. 4a). Thus, based on the functional roles we assign the pathways, the circuit uses a direct pathway decision-space to determine whether to perform an action and an indirect pathway decision-space to determine whether to refrain from it. dsSPNs influence the direct pathway decision-space while isSPNs influence the indirect pathway decision-space, and dmSPNs promote actions while imSPNs discourage actions (Figs. 4a–e**, Modeling time-variant input,** Methods). The circuit uses these compartment-specific mechanisms to calculate which actions *should* be performed with one set of decision-dimensions and calculate which actions *should not* be performed with another. The answers to these questions might overlap. For instance, the direct-pathway and indirect-pathway space should provide divergent answers to the value of consuming cocaine based on the dimensions they prioritized; the former, focusing on reward, might assign it great value while the latter, focusing on cost, might assign great value to not consuming it. When balances between the direct versus indirect pathways and striosome versus matrix changes, this calculus changes. For example, it has been found that dopamine release is enhanced to sSPNs versus mSPNs after cocaine administration^45^ and simultaneously dSPNs are enhanced in the short-term and iSPNs over longer-term scales of time^46^. This might lead to a high-dimensional direct-pathway decision-space but pruning of ordinarily important decision-dimensions from the indirect pathway decision-space, producing a reduction of nuance in determining when to avoid actions and heightened impulsivity.

**Extended Data Fig. 6:**
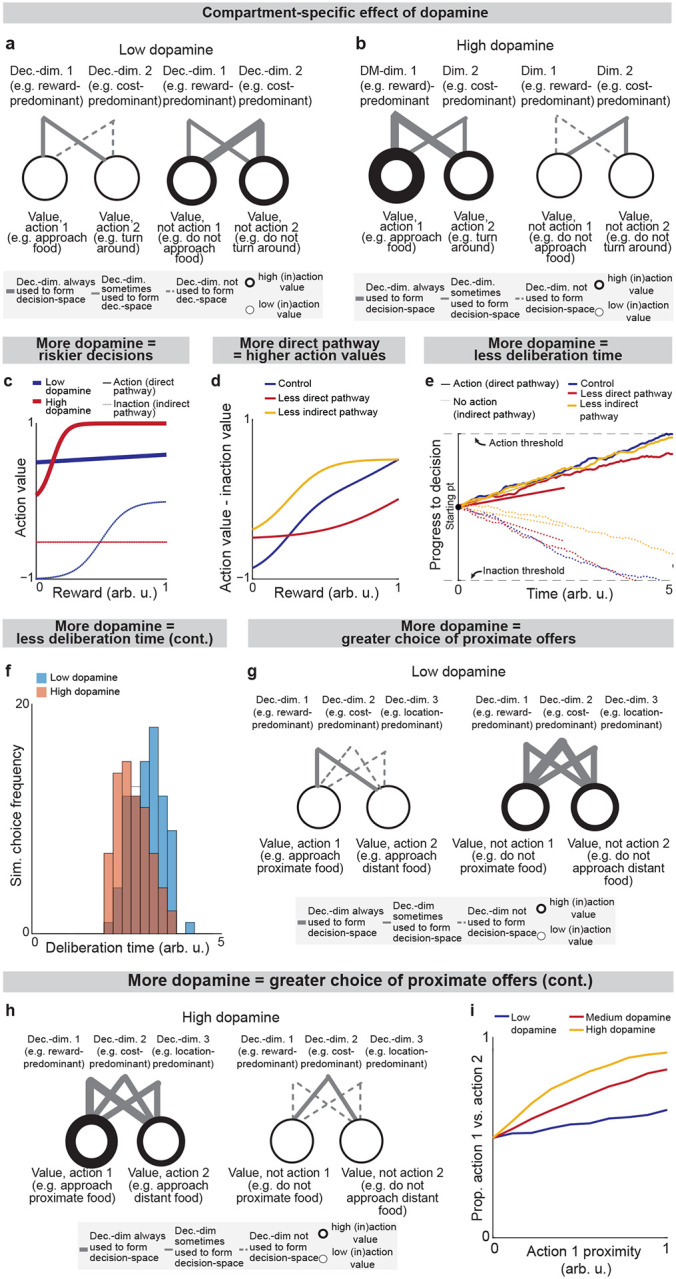
Roles of the direct and indirect pathways, Related to Fig. 4. Here, analysis is conducted using the instance of the model described in Extended Data Fig. 1. **a,b**, Effect of low (**a**) versus high (**b**) dopamine release on decision-space formed by the direct pathway (left panels) or indirect pathway (right panels). When dopamine release is low (**a**), low-dimensional direct pathway decision-spaces are constructed by dsSPNs and high-dimensional indirect pathway decision-spaces are constructed by isSPNs. The opposite happens when there is high dopamine (**b**). To analyze these effects, analysis in Fig. 5 uses an instance of the model where the circuit elements interact dynamically, represented mathematically through a system of differential equations, where sSPN activity for a given decision-dimension i and pathway $P,s_{i,P}(t)$, respond to cortical input after normalization by FSI, $x_{i,P}(t)$, based on weights w between sSPNs, mSPNs, and daSNC elements, a decay factor τ, and a coefficient that controls sSPN→daSNC plasticity, κ (eqs. (13),(14),(15),(16)). The weight of a decision-dimension in mSPN, $S_{i,P}(t)$, occurs dynamically depending on whether or not dopamine release is above a specified threshold (eq. (17)). See ***Instance 3: full connectivity and dynamics***, Methods. In the current figure, we conduct computational analysis using ***Instance 3***, as defined in Extended Data Fig. 1. **c**, Modeled action values for the cost-benefit conflict task where an incremented reward (from a low reward of 0 to a large reward of 1 arb. u.) is accompanied by a constant cost (set to 0.25 arb. u.). Increased dopamine leads to increased action value of high-reward, high-cost options and decreased “inaction value,” thus leading to more approaches when there is high reward and high cost. **d**, The effects of the direct versus indirect pathways are examined in the cost-benefit conflict task used in Figs. 2 and 3. Modeled action values change when dmSPN and dsSPN or imSPN and isSPN are inactivated during a task with incremented reward (from a low reward of 0 to a large reward of 1 arb. u.) with medium cost (set to 0.5 arb. u.). Inactivated dmSPNs and dsSPNs lead to lower action values and inactivated imSPNs and isSPNs lead to larger action values. **e,f,** Modeled deliberation time for the cost-benefit conflict task is shorter when there is more dopamine. Choice is modeled from action values calculated in the cost-benefit conflict task (here, reward = 1 arb. u., cost = 0.5 arb. u.). The method to derive choice from action values (upward-sloping drifts) and inaction values (downward-sloping drifts) is shown in **e** and modeled deliberation times are shown in **f.** **g-i,** The model predicts that dopamine biases actions that contain rewards in physical and/or conceptual proximity. Decision-dimensions important for some decisions but not all will be used to derive action values when there is high dopamine (**g**) but not when there is low dopamine (**h**). If these decision-dimensions correspond to information about location, for instance, then additional dopamine may lead to the incorporation of spatial information in decisions, leading to actions containing the same location information having more similar action values (**i**). (13) $\tau \cdot \frac{ds_{\text{sSPN},i,P}(t)}{dt}=-s_{\text{sSPN},i,P}(t)-x_{i,P}(t)-w_{\text{daSNC}\to \text{SPNN},i,P}\cdot \left(y_{\text{sSPN},i,P}(t)-\frac{1}{2}\right)$ (14)$\tau \cdot \frac{ds_{\text{mSPN},i,P}(t)}{dt}=-s_{\text{mSPN},i,P}(t)+x_{i,P}(t)+w_{\text{daSNC}\to \text{mSPN},i,P}\cdot \left(y_{\text{sSPN},i,P}(t)-\frac{1}{2}\right)$ (15) $\frac{d}{dt}w_{\text{sSPN}\to \text{daSNC},i,P}(t)=\kappa \cdot s_{\text{sSPN},i,P}(t)$ where: (16) $y_{\text{sSPN},i,P}(t)=\frac{1}{1+\text{exp}\;\left(w_{\text{sSPN}\to \text{daSNC},i,P}(t)\cdot s_{\text{sSPN},i,P}(t)+\text{RMTg}-z_{\text{daSNC},i,P}\right)}$ (17) $S_{i,P}(t)=\left\{\begin{array}{ll} 0 & y_{i,P}(t)<\text{threshold}\\ 1 & y_{i,P}(t)\geq \text{threshold} \end{array}\right.$

Indeed, the model’s interpretation of the two parallel decision-spaces offers an intuitive explanation for a range of experimental observations on the direct versus indirect pathway. For example, our model replicates the experimental observations that increased dopamine leads to riskier and quicker decisions and preference for nearby offers, in time or physical proximity, compared to distant offers (Extended Data Fig. 6, Table 8**, Effect of dopamine on action/inaction values** and **Effect of decision-dimensions on choice,** Methods).

### Prediction error encoding is an emergent property of the model.

A range of experimental studies have shown that SPN activities track prediction errors^12,47^. This observation has led to hypotheses that SPNs encode a function related to prediction errors in a reinforcement learning framework^12^. The decision-space model offers a different explanation. In our model, the weights from cortical neurons to SPNs naturally separate cortical information by their associations. For instance, if a bell tends to sound when a subject drinks chocolate milk, both stimuli, even if they arrive from different cortical sources, will likely be mapped to the same reward-related decision-dimension as synapses adjust per Oja’s rule. Less reliable cues are expected to develop mappings with smaller weights. Therefore, the activities of reward-related SPNs may rise when cues predicting rewards appear and fall when cues predicting less reward appear. A sudden change to reward information, for instance a predictive cue, should thus lead to a sudden change in the activity of an SPN subpopulation related to reward, and this change in activity should resemble a prediction error (Figs. 4f–h). Thus, in contrast with the more traditional interpretation where SPNs internally encode a temporal difference value function, the decision-space model suggests that the mapping of cortical activity to the basis of striatal decision-dimensions is sufficient to track prediction error in many cases, without additional computational work performed by the SPNs.

Both interpretations can be used to explain much of the experimental evidence, although the interpretation of the decision-space model may more closely align to recent experimental data (Tables 3,4). Our model also may provide a functional rational for the observation that separate SPNs encode data along different informational axes^12,14^. In fact, our model expects more of these axes to be uncovered by future work. We might expect, for instance, a cue predictive of the novelty of an object to produce an immediate change in activity of an SPN subpopulation related to novelty.

How might the circuit respond in cases where new information diverges sharply from expectations? Our model predicts that in these cases, sSPNs will signal to daSNC that a decision-dimension should be reprioritized, effectively adding or removing the dimension from the decision-space. Interestingly, the circuit has an inherent physiological mechanism to quickly transition away from a decision-space that is no longer optimal. Experimental evidence has identified rebounds in daSNC activity^21^ and striatal dopamine release^18^ after sSPN optogenetic stimulation. Our model suggests that these observations are part of a system by which the circuit can rapidly de-prioritize a decision-dimension after a negative prediction error (e.g. less reward than expected). Thus, the circuit is able to quickly shift to a more helpful decision-space (Fig. 4i).

## Discussion

We found evidence, through the experimental literature and our analysis of neural recordings, to support our hypothesis that modeled physiological patterns in SPN activity (the decision-space) can be used to predict patterns in decision-making, and vice versa. This supports our model of the roles of striosomes and matrix neurons of the direct and indirect pathways in context-dependent decision-making.

**Extended Data Fig. 7:**
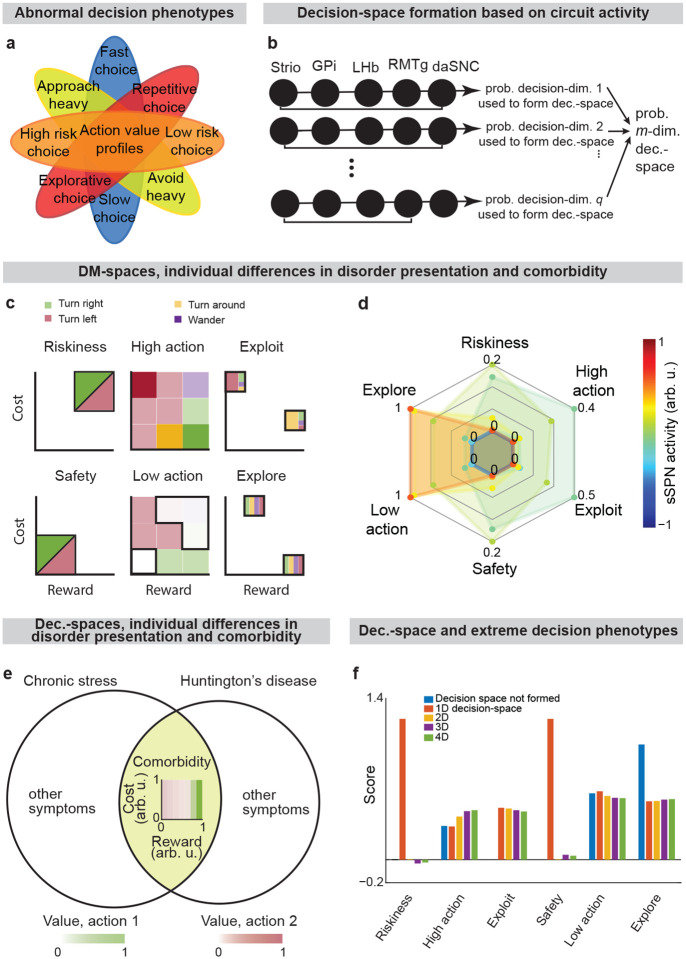
**Individual decision-making differences can be explained by differences in decision-space, Related to** Fig. 5. **a**, Decision-making symptoms observed in disorders. **b**, Schematic of the computational model for simulating decision-space based on circuit activity in the analyses plotted in Figs. 7a,b. daSNC activity ${\text{daSNC}}_{1}={\text{daSNC}}_{2}=\ldots ={\text{daSPN}}_{q}=d$ is calculated for each of q elements, each corresponding to a decision-dimension. Decision-space dimensionality is determined from the Poisson binomial distribution of individual decision-dimension probabilities, per eq. (18). See **Defining mSPN activity and decision-space,** Methods. **c**, Illustration of the method by which we score subjective valuations along six axes (riskiness, safety, high action, low action, exploit, explore) for a modeled T-maze task. A grid of action values for a range of reward and cost combinations is formed. The “riskiness” and “safety” axes are calculated based on the action values for the high-reward high-cost combinations and low-reward low-cost combinations, respectively. “High action” and “low action” axes are calculated as the proportion of the reward/cost grid with especially high (sum of all action values > 0.5) or low (< 0.2) action value, respectively. The “exploit” and “explore” axes are determined as the proportion of the reward/cost grid with especially high (> 0.5) or low (< 0.25) Gini coefficients between the action values. **d**, Extension of **Fig. 7c.** sSPN activity is modified, leading to decision-spaces formed at different rates. These different decision-spaces lead to different action valuations. Thus, in a disorder which affects sSPN activity during decision-making, differences in decision-space may be responsible for differences in decision-making. **e**, Huntington’s disease and chronic stress both have decision-making signatures of low-dimensional decision-spaces. **f**, Extension of **Fig. 7f,** showing that scores along the six subjective valuation axes (mean taken over 1000 simulations) are different depending on decision-space. Thus, disorders that affect decision-space formation may lead to shifts in decision-making. (18) P(mDM-dimensionsusedtoformDM-space)=qmdm(1-d)q-mform=0,1,…,q

**Fig. 5:**
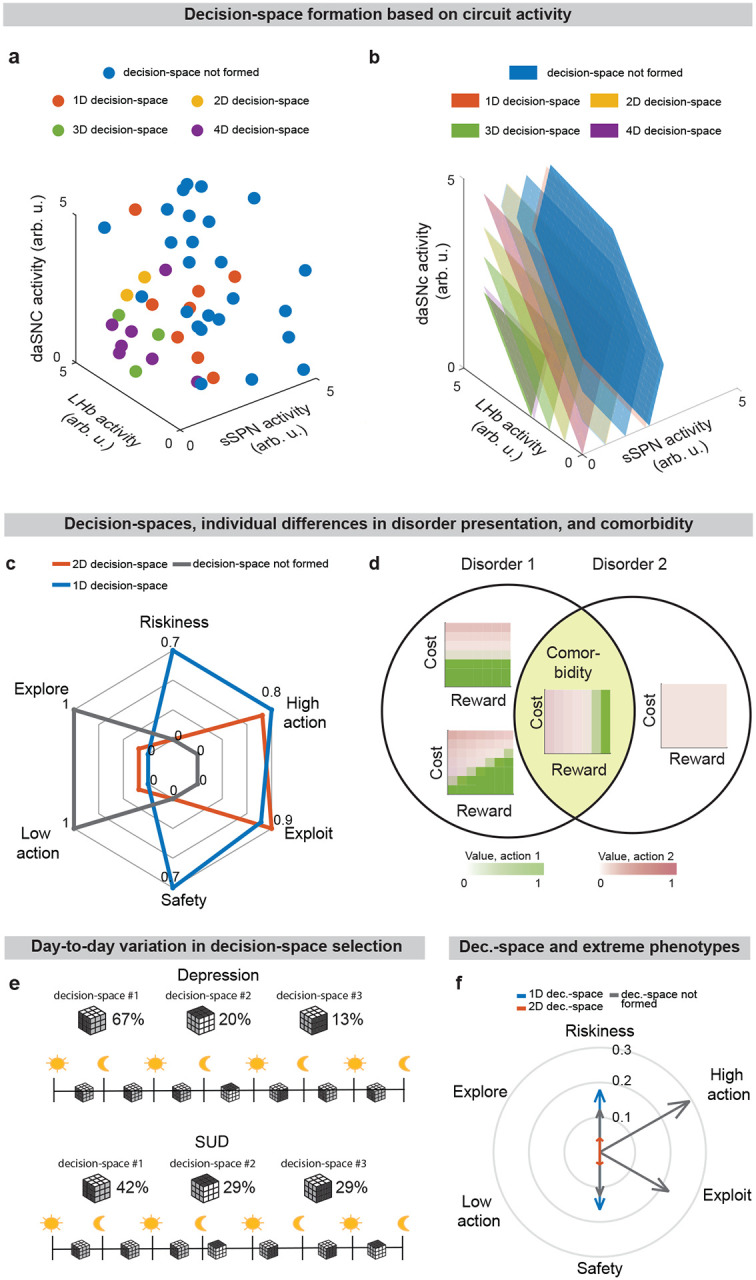
Differences in decision-space could explain comorbidity, individual differences, and daily variations. **a**, A decision-space is simulated for various sSPN, daSNC, and LHb activities. A high-dimensional decision-space (yellow, green, purple points) tends to form when sSPN activity is low, daSNC activity is high, and LHb activity is low, although there is an element of probability. **b**, The probabilities of the decision-spaces in a are shown using isosurfaces. Three isosurfaces are displayed for each decision-space dimensionality (probability = 0.25, 0.5, and 0.75 of formation, from lightest to darkest). The circuit forms a bias towards certain decision-spaces over others, but different decision-spaces can form at the same circuit activity. **c**, Summaries of overall decision-making profiles across trials in a T-maze task, scored in six ways, showing that transitions between decision-spaces can lead to very different decisions. A disorder could produce a bias, for instance, towards low-dimensional decision-spaces, which would in turn alter the decision-making profile. Scores are formed by quantifying the trend of action values across reward and cost levels. Riskiness/safety: treatment of high-reward, high-cost (or low-cost, low-reward) levels; high/low action: tendency towards high (or low) action values; exploit/explore: tendency to focus on one action versus many (see Extended Data Fig. 7c). **d,e,** Differences in circuit activity between individuals could lead to decision strategies observed at different rates, as is the case in individuals with disorder comorbidity (**d**). Further, day-to-day shifts in circuit activity shifts could cause stark differences in decision strategies between days (**e**). Cartoon shows the hypothetical use of different decision-spaces for depression and substance use disorders. **f**, Certain decision-spaces more often lead to action values that are extreme (ratio formed over 1000 simulations, as scored using the metrics in **c**), a feature of disorders. Vector length corresponds to outlier rate (proportion of scores for each group that fall within the top 10% of observations across all groups).

Due to the circuit’s important role in decision-making processes including in neuropsychiatric disorders, our model provides a framework with which to study decision-making phenomena commonly observed in psychiatry. An important prediction of our model is variance in context-dependent decisions, between individuals and over time (Figs. 5a–e, Extended Data Fig. 7a–e**, Effects of sSPN, LHb, and daSNC activity on choice profiles,** Methods). Individual differences in decision-making as a function of disorders, as seen in the experimental literature^48–50^, could arise in cases where there are slight differences in activity of the circuit we model, leading to similar decision-making phenotypes only when a similar decision-space is formed. Daily variance in decision-making, a common observation in pscyhology^51^, could arise from daily variance in circuit activity, causing daily variance in the decision-spaces formed most often. Further, differences in circuit activity may explain the established inter-individual differences in the severity of psychiatric disorder symptoms observed during decision-making^52,53^. Individual differences in disorder susceptibility could arise from reliance upon or avoidance of a decision-space that leads to extreme decision-making tendencies (e.g. extremely action-heavy, extremely risk-averse) when combined with abnormal action value rules in mSPNs (Fig. 5f and Extended Data Fig. 7f).

**Extended Data Fig. 8:**
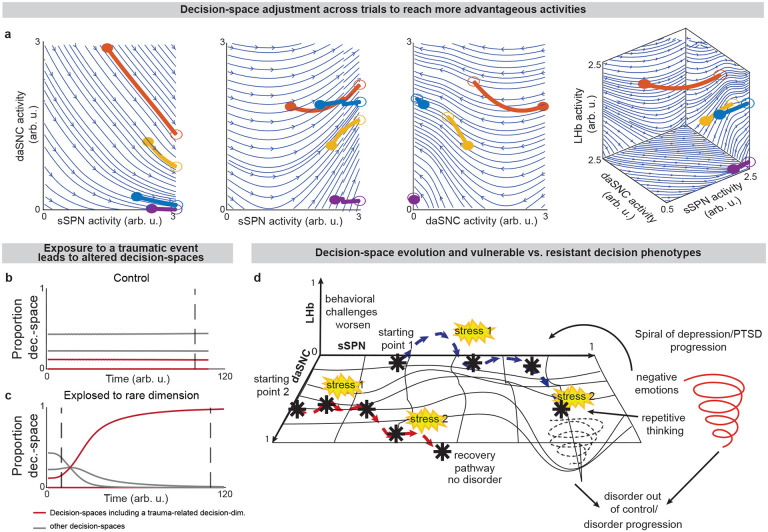
**Disorder development and decision-space formation, Related to** Fig. 6. **a**, Simulations of trajectories of circuit activity between trials. Four trajectories of circuit activity movement (thick lines) are plotted above streamlines (blue lines) for a modeled circuit that adjusts to facilitate simple choices. Filled circles are various initial circuit activities ($t_{0}$). Empty circles are the ending circuit activities (i.e. $t_{t}$). The left three panels are two-dimensional slices of the plot on the right. Here and in Fig. 6, the modeled circuit adjusts between trials to improve its ability to form preferred decision-spaces (advantage) while avoiding large changes to activity during decisions (cost). Advantage is represented mathematically as a weighted sum of probabilities that the possible decision-spaces form, given the circuit elements $\left\{X_{1},X_{2},\ldots ,X_{n}\right\}$ take a specified set of activities $x_{1},x_{2},\ldots ,x_{n}$ (eq. (19)). Cost is represented as the distance between the circuit activity used during decision-making and a “baseline” circuit activity that the circuit takes outside of decision-making (eq. (20)). Net advantage is the difference between advantage and weighted cost (eq. (21)). The circuit adjusts between trials in the direction of maximal change in net advantage (eq. (22)). See ***Movement of Circuit Activity Across Multiple Trials,*** Methods. **b,c,** A possible explanation for the observation that in certain psychiatric disorders (post-traumatic stress disorder and substance use disorder) exposure to a traumatic event or drug can lead to increasingly altered choice after an extended period of nonexposure or abstinence. This can be modeled as adaptation in the circuit to process a rare decision-dimension that was necessary to process, for example, the traumatic event or drug. In the simulation, the circuit’s preference for various decision-spaces is updated over time based on their success in making choices according to environmental stimuli. In the disorder resilient scenario (**b**), the circuit is first exposed to the traumatic event or drug at the dashed line. The “red” decision-space is not formed as often as are other decision-spaces (gray lines). In contrast, even short periods of exposure to the traumatic event or drug can impact future decision-spaces, resulting in vulnerability (**c**). Circuit activity adjusts until, when the traumatic event or drug reappears, the “red” decision-space forms frequently. This result may explain incubation of fear (in post-traumatic stress disorder) or craving (in a substance use disorder), where symptoms emerge only after a period of weeks after exposure. Differences in response post-incubation lead to modeled vulnerability or resilience. **d**, Circuit activity morphs over time in response to decision-making needs. (19) $\text{advantage}\left(X_{1}=x_{1},X_{2}=x_{2},\ldots ,X_{n}=x_{n}\right)=\sum_{l=1}^{2q}\;{\text{score}}_{l}\cdot P\left({s\text{pace}}_{l}|\left(X_{1}=x_{1},X_{2}=x_{2},\ldots ,X_{n}=x_{n}\right)\right)$ (20) $\text{cost}\left(X_{1}=x_{1},X_{2}=x_{2},\ldots ,X_{n}=x_{n}\right)={\left\|{\left[\begin{array}{llll} x_{1} & x_{2} & \ldots & x_{n} \end{array}\right]}^{⊤}-{\left[\begin{array}{llll} x_{1,\text{baseline}} & x_{2,\text{baseline}} & \ldots & x_{n,\text{baseline}} \end{array}\right]}^{⊤}\right\|}_{2}$ (21) $\text{net}\;\text{advantage}\left(X_{1}=x_{1},\ldots ,X_{n}=x_{n}\right)=\text{advantage}\left(X_{1}=x_{1},\ldots ,X_{n}=x_{n}\right)-\text{constant}\cdot \text{cost}\left(X_{1}=x_{1},\ldots ,X_{n}=x_{n}\right)$ (22) $\frac{\Delta {\left[\begin{array}{llll} x_{1,\text{baseline}} & x_{2,\text{baseline}} & \ldots & x_{n,\text{baseline}} \end{array}\right]}^{⊤}}{t\text{rial}}=\text{rate}\cdot \nabla \text{net}\;\text{advantage}\left(X_{1}=x_{1},\ldots ,X_{n}=x_{n}\right)$

**Fig. 6:**
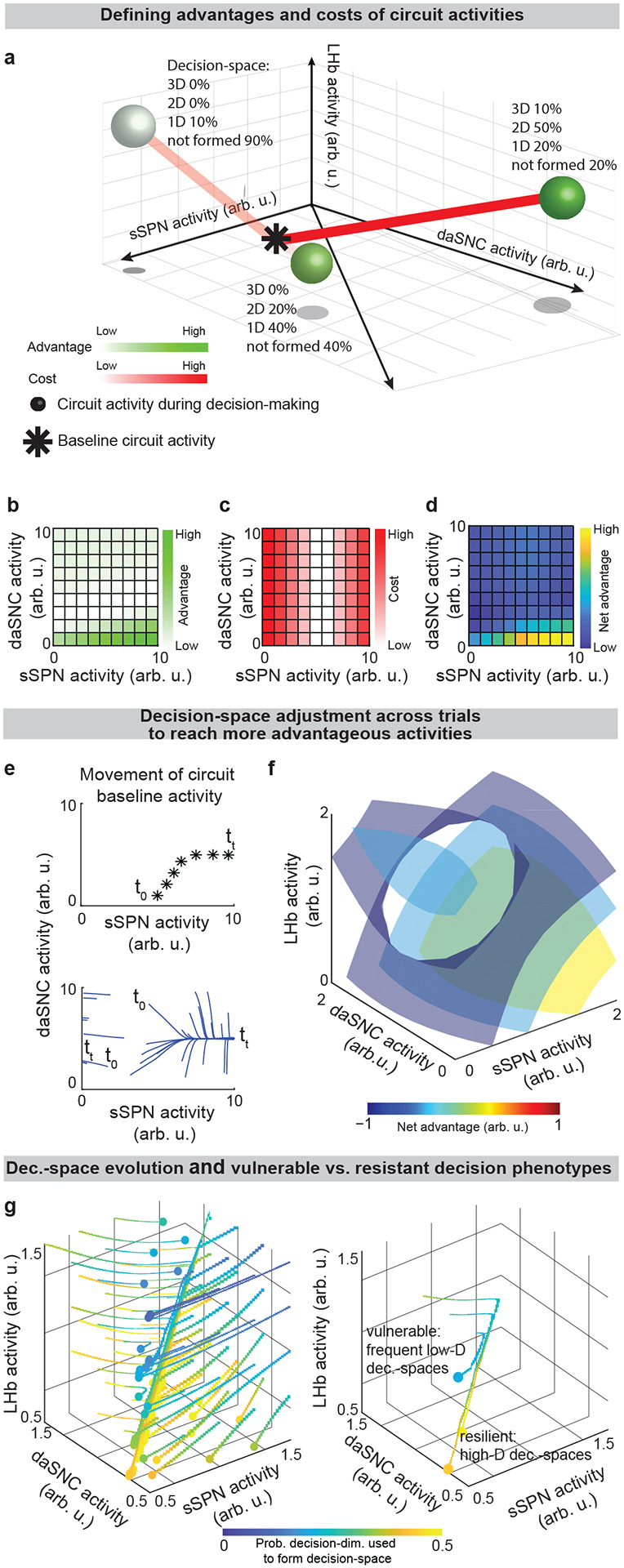
Circuit adapts between trials to form preferred decision-spaces, leading to disorder progression. **a**, Cartoon illustrating how the modeled circuit adjusts to facilitate construction of preferred decision-spaces (“advantage”) while limiting large changes in circuit activity during decision-making (“cost”). Possible advantages and costs (whose difference is “net advantage”) are shown at three circuit activities (balls). The decision-space is formed differently at each circuit activity (annotations), leading to differences in advantage. Between trials, the circuit adjusts its baseline activity (i.e. resting activity outside decision-making) in the direction of highest net advantage. **b-d,** Simulated advantages (**b**), costs (**c**), and net advantages (**d**) shown across sSPN and daSNC activities. **e**, sSPN and daSNC activity adjusts between trials to best form preferred decision-spaces. Due to this, the circuit adjusts its activity from an initial baseline activity $\left(t_{0}\right)$ to other activities associated with the required decision-spaces $\left(t_{t}\right)$. **f**, Similar to **d** but for sSPN, LHb, and daSNC activities, showing a simulated circuit where it is most advantageous to have high sSPN activity during simple choices. The trend in the continuous 3D decision-space is visualized using isosurfaces. **g**, Similar to **e** but for sSPN, LHb, and daSNC activities. Dots show ending circuit activities (i.e. $t_{t}$) and beginnings of lines possible initial circuit activities (i.e. $t_{0}$). Depending on initial activity, the circuit may increasingly use or disregard a decision-dimension when forming decision-space. Right panel shows trajectories from three starting circuit activities, two of which lead to the decision-dimension commonly being used to form decision-space (e.g. resilient subjects) and one which leads to it commonly being disregarded (e.g. vulnerable subjects).

Further, our model serves as a framework for forming hypotheses about changes to the circuit across days and weeks, including during neuropsychiatric disorder progression. Our model expects the circuit to adapt between trials as it adjusts to more frequently form a preferred decision-space (Figs. 6a–f, **Effect of initial circuit activity on future trials**, Methods). So, vulnerability or resilience to disorders can be framed as an adaptation that is favorable (e.g., to adeptly form a high-dimensional decision-space) or maladaptive (e.g., to only form a low-dimensional decision-space, regardless of decision context). Depending on its initial activity, a modeled circuit can adapt to reach very different activity, leading to disposition to either a high- or low-dimensional decision-space (Fig. 6g and Extended Data Fig. 8a). Thus, differences in the circuit before exposure to a traumatic event, for instance, may explain why two subjects that encounter the same traumatic event do not always develop the aberrant decision-making symptoms of PTSD^54^. It may also shed light on the neural processes underlying incubation of fear^55^ and incubation of craving^56^, where disorders progress over the span of weeks or months, even when the traumatic event or addictive substance does not reappear (Extended Data Figs. 8b–d**, Effect of altered advantage score on future trials,** Methods). By representing the role of SPNs in a compartment-specific way, our model facilitates understanding of disorders that affect striosomes and matrix differentially.

Our model adds detail to the predictions made by a range of other models of basal ganglia function. For example, a range of other models consider how the direct and indirect pathways of the basal ganglia interact to moderate action selection^25,57^. Our model offers a simple explanation for several experimental phenomena that are used to fit other models by differentiating the roles of striosomes versus matrix (Extended Fig. 6). In particular, direct/indirect pathway models more commonly fit data from simple reward and/or cost tasks rather than highly difficult tasks, and our model allows for scaling to difficult tasks with many informational dimensions. Another class of model examines how RPE signals facilitate adaptability^58–60^. Our model does not preclude the possibility that SPNs and/or dopamine may encode an algorithm similar to those used in reinforcement learning. However, our model expects that such an algorithm would function within the framework of the decision-space, perhaps by encoding a separate reinforcement learning value function along each decision-dimension, instead of a traditional model that learns only the discounted sum of future rewards. Exploring this possibility would add depth to current studies of reinforcement learning in the basal ganglia. Our model can additionally be used to expand on other models that explore the role of basal ganglia pathways in performing dimensionality reduction^61^ or responding to events as sequences that influence each successive action^62,63^. In each of these cases, our model adds detail on the functional process by which the basal ganglia prepares for a decision based on context. It also clarifies the mechanisms by which the compartments of the striatum process information, led by striosomal influence on dopamine.

**Extended Data Fig. 9:**
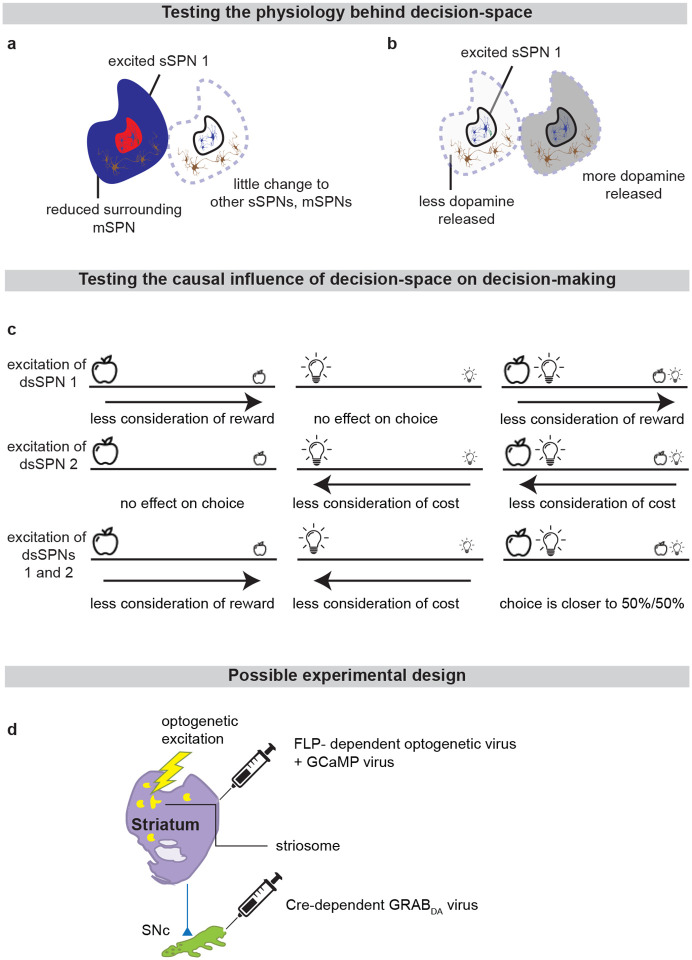
Proposed experiments that might add support to our model. **a**, The decision-space model assumes that when an sSPN subpopulation is active, the corresponding decision-dimension is unlikely to be used to form the decision-space. Thus, activating an sSPN subpopulation (red) should lead to lower activity in neighboring mSPNs (blue) and there should be lower variance in their activities. This would support our hypotheses that sSPNs bias a nearby mSPN subpopulation towards being used or excluded during the formation of decision-space. **b**, Meanwhile, we would expect to find less dopamine (light gray versus darker gray) released to the sSPN subpopulation and neighboring mSPNs than to other SPNs. This would support our hypothesis that the decision-space is formed via selective dopamine release. **c**, We would expect sSPN stimulation to alter decision-making. An experiment could be designed asking rodents to choose rewards (apple) and/or costs (lights) in a T-maze (line, animal begins in the center). The rodents would be expected to selectively deprioritize informational dimensions corresponding to sSPN subpopulations that are stimulated. For instance, stimulating reward-responding sSPNs would be expected to lead to reduced consideration of reward when making the decision. **d**, The hypotheses in **a-c** can be tested with tools available to neuroscientists. Similar to was performed by Lazaridis et al. (2024), DA-Cre-expressing mouse are crossed with striosomal-FLP-expressing mice. Three viruses are injected: 1) Cre-dependent ${\text{GRAB}}_{\text{DA}}$ into daSNC (for instance, similar to Sun et al. (2020))^81^, 2) FLP-dependent optogenetic virus into the striatum, 3) general G-CAMP virus into the striatum. Striosome and matrix are then recorded using two-photon microscopy, similar to, for example, Bloem et al. (2022). Four colors are used to: 1) identify dopamine via the ${\text{GRAB}}_{\text{DA}}$ virus 2) identify all striosomal and matrix neurons via the GCaMP virus, 3) identify the contours of the striosomes via the FLP-dependent virus, and 4) stimulate the striosome. In a closed loop way, striosome neurons are identified that correspond to reward or cost, and the neurons that selectively respond to each are stimulated as rodents perform a T-maze task that was used by, for instance, Friedman et al. (2015).

Our model carries several limitations, including its limited focus on a dorsomedial striosomal circuit and certain physiological assumptions (Supplemental Notes 1–4). We limit our focus to a specific circuit that has been implicated in decision-making, rather than attempt a unifying theory of basal ganglia function or decision-making encoding across the brain (Supplemental Note 1). While we demonstrate alignment to the existing experimental evidence in Figs. 2 and 3 and Tables 3–8, future experiments (outlined in Extended Data Fig. 9) will be required to confirm the several assumptions we make. Despite these limitations, our model has demonstrated success in relating neural activity to decision-making across a range of behavioral tasks and has the power to explain a range of phenomena, from neural processes to psychiatric observations.

## Extended Data

## Supplementary Material

1

## Figures and Tables

**Table 1:** Terminology

Term	Definition
**GPi**	Globus pallidus internus
**GPe**	Globus pallidus externus
**LHb**	Lateral habenula
**RMTg**	Rostromedial tegmental nucleus
**daSNC**	dopaminergic neurons of the substantia nigra compacta
**FSI**	Striatal fast-spiking Interneuron
**sSPN**	Striosomal striatal projection neuron
**mSPN**	Matrix striatal projection neuron
**dsSPN**	Direct pathway sSPN
**isSPN**	Indirect pathway sSPN
**dmSPN**	Direct pathway mSPN
**imSPN**	Indirect pathway mSPN
**BG**	Basal ganglia
**Decision-dimension**	An axis of the coordinate system with which SPNs (dsSPNs, isPSNs, dmSPNs, imSPNs) process cortical activity. Subpopulations of SPNs (for each dsSPNs, isPSNs, dmSPNs, imSPNs) encode data along different decision-dimensions. Cortical activity is linearly mapped to this basis of decision-dimensions such that the activity of a single cortical neuron no longer is encoded by a single SPN. Certain decision-dimensions might correspond more predominantly, for instance, to reward level, cost level, or novelty level, as encoded across multiple cortical neurons. Decision- dimensions are modeled as the principal components of cortical activity.
**Decision-space**	The subspace produced by the decision-dimensions which are selected by the circuit to be used during a decision. Decision-space is formed when dopamine releases to mSPNs (dmSPNs and imSPNs), signaling that certain decision-dimensions are important and others are unimportant, and therefore can be excluded from the subspace.
**Action value**	Value assigned by the circuit to an action
**Inaction value**	Value assigned by the circuit for refraining from an action
**Prediction error**	The difference between expected and observed information along a data axis (for instance, reward prediction error, punishment prediction error, or novelty prediction error).
**Circuit activity**	The set of average activities of each circuit element during a decision
**Advantage**	The degree to which a circuit activity is preferred. Used in our analysis of the change in circuit activity between trials.

**Table 2:** Evidence for the connectivity used in our model.

Connectivity	Evidence
cortexis→SPN/mSPN	Research has found that nearly, if not all cortical regions project to the striatum^64,65^, however only a subset of cortical areas have been determined to be compartment specific^4,41^, with examples of those listed below. Evidence that both the striosomes and matrix receive input from sensorimotor, limbic, and associative regions^66^. Evidence that several regions (prelimbic cortex, infralimbic cortex, posterior orbitofrontal cortex, insular cortex) project more to striosomes than matrix^2,67^. Evidence that several regions (primary motor cortex) project more to matrix than to striosomes^68^.
cortex→FSI→sSPN/mSPN	Evidence for FSI connections to both striosomes and matrix^3,10,11^.
sSPN (more so than mSPN)→daSNC	Range of evidence from primates, rats^24,69^, and mice^68^ suggesting a stronger connection striosome connection than matrix. One study suggests that matrix also projects to daSNC^70^.
sSPN (more so than mSPN)→GPi→LHb→RMTg→daSNc	Range of evidence supporting the striosome to EP (non-primate GPi correlate) connection in rats^23,24^. Evidence supporting the striosome to GPi connection in primates^71^. Evidence of the EP to LHb connection in rats^72^. Evidence that striosomes more so than matrix drive striatal influence on LHb activity^22^, potentially through pallidal regions^73^. Evidence in primates^72^, rats^74^ , and mice^73^ of the LHb to RMTg (also called tVTA) connection. Evidence that the RMTg projects to daSNc^75,76^.
sSPN (more so than mSPN)→GPe→daSNC	Evidence of the striosome to GP (non-primate GPe correlate) but not matrix to GP connection in mice^16^ and rats^24^. Evidence of a striosome (more so than matrix) to GPe to daSNC pathway^16^.
daSNC→sSPN/mSPN	Evidence that dopamine is released to both compartments, with faster^77^ and more^8,45^ dopamine release to striosomes than matrix.

**Table 3:** Criteria used in Tables 4–8 to test our decision-space model and compare it to alternative models of the circuit.

Modeled Functional role of a circuit region.	Expectation per the model.
**sSPNs influence the priority of decision-dimensions, thereby affecting decision-space.** **(decision-space model)**	**1.A.** During a high-dimensional decision-space, choice more closely aligns to experimental inputs (e.g. chocolate milk level, light brightness) during difficult tasks (e.g. consideration of both rewards and costs).
	**1.B.** A low-dimensional decision-space is often formed from decision-dimensions which are commonly used during a decision-making task, for instance information about reward in rodents that are trained to respond to reward cues.
	**1.C.** Cortical data is mapped orthogonally and continuously onto SPNs.
**sSPNs encode conflict.** **(our conflict model)**	**2.A.** sSPN activity scales with conflict between two features like reward and cost.
	**2.B.** Changes to conflict, revealed by sSPN activity, alter choice.
	**2.C.** Changes to sSPN signals are greatest when conflict is introduced.
**sSPNs encode subjective values.** **(our subjective value model)**	**3.A.** sSPN activity scales (possibly directly or inversely) with subjective value of stimuli, likely roughly tracking reward minus cost.
	**3.B.** Higher subjective value, reflected by sSPN activity, leads to increased selection of an offer.
	**3.C.** Changes to sSPN activity are strongest at the time during scenarios where cues are associated with subjective values.
**sSPNs encode prediction errors.** **(our prediction error model)**	**4.A.** sSPN activity should change proportionally and continuously to the difference between expected and actual reward and cost at each time step.
	**4.B.** As a task is learned, the trend in sSPN activity over time changes as earlier cues become associated with later outcomes.
**sSPNs encode actions.** **(our actions model)**	**5.A.** Different neurons would encode different actions
	**5.B.** Activity of sSPN subpopulations should scale directly or inversely with the predisposition of action execution.
	**5.C.** Changes to the signals of action-encoding subpopulation would be greatest prior to or during action execution.
**GPi during decision-space formation.** **(decision-space model)**	**6.A.** Changes to activity along a decision-dimension is reflected in GPi activity.
	**6.B.** A given GPi neuron encodes data across multiple decision-dimensions.
	**6.C.** Activation of GPi causes more decision-dimensions to be incorporated into the decision-space while inactivation causes fewer decision-dimensions to be used.
**LHb and RMTg optimizing or modifying decision-space.** **(decision-space model)**	**7.A.** Active LHb (or RMTg) leads to choice reflective of reduction in dimensionality of the decision-space and vice versa.
	**7.B.** LHb (or RMTg) is active during times when lower-dimensional decision-spaces are beneficial to decision-making.
**Dopaminergic neurons of the SNc during decision-space formation.** **(decision-space model)**	**8.A.** There exist subpopulations of daSNC neurons that encode information along an orthogonal axis of information.
	**8.B.** Activity in one daSNC subpopulation only affects the subpopulation of SPNs corresponding to one decision-dimension.
**Direct and indirect pathways alter decision-space formation.** **(decision-space model)**	**9.A.** Higher dopamine leads to lower dimensionality of the direct pathway decision-space while lower dopamine leads to higher dimensionality, and vice versa for the indirect pathway.
	**9.B.** Direct pathway mSPNs promote actions while indirect pathway mSPNs aid in action suppression.
	**9.C.** Subpopulations of mSPNs encode data along decision-dimensions orthogonally and continuously over time.

**Table 4:** Testing alignment of the decision-space model and other models to a selection of the experimental sSPN literature.

	Criterion	Friedman et al. (2015)^11^ (I)	Friedman et al. (2020)^10^ (II)	Bloem et al. (2022)^12^ (III)	Xiao et al. (2020)^14^ (IV)	Weglage et al. (2021)^13^ (V)
Decision-space model	1.A	✓	✓	∅	≈	∅
	1.B	∅	✓	∅	∅	∅
	1.C	∅	∅	✓	✓	✓
Conflict model	2.A	✓	✘	✘	✘	∅
	2.B	✓	∅	∅	∅	∅
	2.C	✓	x	✘	✘	ϕ
Subjective value model	3.A	✘	✓	✘	✘	✘
	3.B	✓	✓	∅	✓	✘
	3.C	✓	✓	✓	✓	✘
Prediction error model	4.A	✘	✘	✓	✘	✘
	4.B	∅	✘	✓	✓	∅
Actions model	5.A	∅	∅	✓	✓	≈
	5.B	✓	✓	✓	✓	≈
	5.C	≈	✓	✓	✓	≈

✓ -- aligned with criterion

≈ -- somewhat aligned to criterion

✘ -- not aligned with criterion

∅ -- experiment does not test criterion

**1.A.I.** Striosome activation led to less consistent choice in tasks that required processing of both reward and cost, while sSPN inhibition led to more consistent choice in tasks that required processing of both reward and cost. Decision-making was less affected by sSPN manipulation in tasks that required processing either reward or cost, but not both. Meanwhile, in the absence of manipulation, the rodents had less active sSPNs during tasks that required processing both reward and cost. These results may suggest that a higher-dimensional decision-space (formed at low striosome activity) is associated with processing of multiple informational dimensions in a consistent way.

**1.A.II.** Rodents that best learned the reward/cost cue discrimination task had high sSPN activity after the reward cue and low sSPN activity after the cost cue. The rodents that learned less well had similar activity between the tasks. This may suggest that the most consistent choices, made by the rodents that learned, were formed using a context-dependent decision-space.

**1.A.IV.** Inhibition of Tfz1 neurons, which were shown to encode either reward or cost independently, decreased accuracy and correct rejection rate in cost trials. This may suggest a change to the priority assigned to the reward and cost decision-dimensions. Notably, the direction of the effect is opposite what the model expects, if only cost-related SPNs were inhibited. However, Tfz1 neurons that responded to rewards and costs were inhibited simultaneously, making for a less clear model prediction on the direction of the effect.

**1.B.II.** More rodents successfully learned the reward task than the cost task, and this was reflected in fewer rodents forming a reduced sSPN activity and reaching a putative high-dimensional decision-space. This may suggest that most rodents formed a one-dimensional reward-related decision-space and only the rodents that learned formed, in the appropriate context, a two-dimensional reward-and-cost-related decision-space.

**1.C.III.** In the probabilistic bandit task, dynamic changes along the orthogonal information axes of reward and cost led to proportional changes in sSPNs that resembled prediction errors. Some of the identified neurons responded only to reward or only to cost. This may suggest that those neurons are members of subpopulations corresponding to a reward-predominant decision-dimension or a cost-predominant DM-dimension.

**1.C.IV.** Tfz1-expressing sSPNs showed evidence of encoding reward or cost dimensions independently (although it was not confirmed that Tfz1 neurons were responding to cortical signals). During a real-time place preference task, different neurons were activated by either reward or cost individually both during administration of the reward or cost and at a cue associated with the reward or cost. This may suggest encoding of information along decision-dimensions, including related information observed from separate stimuli.

**1.C.V.** During a multiphase task that required attention to many stimuli and strategies, sSPN and mSPN activities in general followed somewhat similar activity patterns over time. The most significant indicator of SPN activity was task phase. This may suggest a continuous mapping, with similar weights between sSPN and mSPN, of cortical information relevant to the current phase of the task onto the decision-space in the striatum.

**2.A.I.** Mean sSPN activity was significantly different in the task where there was the most conflict than in the other tasks.

**2.A.II-IV.** Conflict was not introduced experimentally, yet sSPN activity was different between tasks.

**2.B.I.** Optogenetic manipulation of striosomes, which may alter the level of encoded cost/reward conflict, altered choice.

**2.C.I.** In the task, conflict is important to the rodent when it determines which option to select, which is when striosome activity rises. This may suggest that striosomes encode conflict.

**2.C.II-IV.** Conflict was not introduced experimentally, yet sSPN activity spiked when non-conflict stimuli were introduced.

**3.A.I.** sSPN activity did not scale, directly (more sSPN activity, more subjective value) nor inversely (more sSPN activity, less subjective value), with either the overall values of the options or with the difference between the value of the options on the right arm of the T-maze versus the left arm. sSPNs had lowest mean activity in the cost-benefit conflict task, which had a moderate difference between reward and cost. Both the benefit-benefit task, which had high reward and no cost, and the cost-cost task, which had low reward and high cost, were performed with high sSPN activities.

**3.A.II.** sSPN activity was higher during the reward task than the cost task among rodents that learned the task. This may suggest that sSPN activity tracks subjective value of the presented stimuli, with the reward-related stimuli being assigned higher subjective value than the cost-related stimuli.

**3.A.III.** The activities of a sizable subpopulation of SPNs were found to track reward level and cost level separately, not together as our subjective value model would expect. Meanwhile, neurons that tracked both reward and cost had activities that did not scale with reward minus cost, suggesting encoding of information other than the subjective value of the stimuli.

**3.A.IV.** Separate SPN subpopulations were found to track reward or cost level, suggesting encoding of reward and cost separately, rather than together as our subjective value model would expect.

**3.A.V.** There was not a strong relationship between rewards minus costs presented in the different task phases and sSPN activity.

**3.B.I.** The rodents approached the higher-reward option most in the task (cost-benefit conflict task) which had the lowest sSPN activity, suggesting a possible relationship between subjective value assigned to the left and right arms of the T-maze and sSPN activity.

**3.B.II.** The choice to lick was made more frequently when sSPN activity during the licking period was higher.

**3.B.IV.** Stimulation and inhibition of Tfz1-expressing neurons led to opposite effects on decision-making, suggesting that manipulation may change subjective values assigned to stimuli.

**3.B.V.** There was not a strong relationship between sSPN activity and choice in the multiphase task.

**3.C.I.** Across all tasks, sSPN activity ramped during the period of the decision where a choice was made as to which offer to approach, which is also likely the period of the task that most requires an assignment of subjective value to environmental stimuli. This suggests that perhaps sSPNs play a role in assigning subjective value.

**3.C.II.** sSPN activity rose during the periods when the rodents chose whether to lick, which is the period of the task that likely most requires an assignment of subjective value to environmental stimuli. This suggests that perhaps sSPNs play a role in assigning subjective value.

**3.C.III.** sSPN activity was the highest at the cue and during the outcome period, two intervals when it is likely important to assign subjective value to stimuli. This suggests that perhaps sSPNs play a role in assigning subjective value.

**3.C.IV.** sSPN activity rose during the cue and administration periods of the Pavlovian conditioning task, both of which are likely important for assignment of subjective value. This suggests that perhaps sSPNs play a role in assigning subjective value.

**3.C.V.** sSPNs did not have exceptionally high nor low activities during periods of the multiphase task in which we might expect subjective value assignment to be important. sSPN activity was higher during a locomotion phase, for example, than during a phase where a reward was presented.

**4.A.I.** The rodents in the experiments were overtrained, task types were randomized, and each task type had different levels of reward and/or cost. Therefore, they might be expected to experience prediction error when they entered the maze and become aware of the task type. Prediction upon entering the maze should be roughly equivalent to the average value (reward minus cost) of all tasks. By this logic, there is positive prediction error in tasks where the expected outcome was greater than the expected outcome upon entering the maze, and vice versa. Thus, the largest positive prediction error likely occurred during the benefit-benefit task, followed by the cost-benefit non-conflict task, followed by the cost-benefit conflict task, followed by the cost-cost task. Experimental sSPN activities were not ordered like this.

**4.A.II.** Theoretically, we would expect, post-learning, a prediction error at the tone because the value of the session changes at this point. Yet sSPN activity had little change at the tone post-learning.

**4.A.III.** The authors demonstrated that separate populations track prediction error directly and inversely.

**4.A.IV.** Theoretically, prediction error moves from the administration period to the cue during a Pavlovian task. This is not what was observed: sSPN activity spiked during the administration period just as much after learning as before.

**4.A.V.** sSPN activity had little change at periods of the task when prediction errors were introduced experimentally.

**4.B.II.** sSPN activity over time did change with learning but spikes in activity did not develop at times (e.g. the cue) we would expect post-learning prediction errors to develop.

**4.B.III.** sSPN activity tracked prediction error more closely at the cue after learning than before. This suggests that sSPNs may be encoding prediction error and learning the association between the cue and the outcome.

**4.B.IV.** The activities of individual SPN increased in magnitude at the cue after learning in the Pavlovian conditioning task (although there was not, as our prediction error model would expect, a corresponding reduction during administration). In the active avoidance task, failure-responding sSPNs increased activity upon punishment delivery.

**5.A.III.** Different neurons were found to respond to reward versus cost. This may suggest that different neurons were involved in encoding actions planned in response to the different stimuli.

**5.A.IV.** Different neurons were active in response to reward versus cost. This may suggest that different neurons were involved in encoding of actions planned in response to the different stimuli.

**5.A.V.** Neurons encoded actions in task-specific contexts but remapped between tasks.

**5.B.I.** The optogenetic manipulation caused altered actions (turn right versus turn left), suggesting that perhaps stimulation or inhibition leads to upweighting of one action versus another.

**5.B.II.** Different actions (licking versus non-licking) corresponded to different mean sSPN activity. This may suggest that sSPNs encode the action of licking.

**5.B.III.** sSPN subpopulations changed in response to unexpected stimuli, perhaps to recalculate actions to take.

**5.B.IV.** The reward-active sSPNs were most active during the outcome period and less so during the cue. This could be a sign of a potential action linked to the stimuli.

**5.B.V.** sSPN subpopulations were identified but their activities were only related to actions on a task-by-task basis.

**5.C.I.** For many trials, sSPN activity increased during the period when the animal made a choice between turning left versus turning right. However, sSPN activity was roughly constant throughout the choice periods of cost-benefit conflict trials, and the actions model would expect a ramping of activity here, too.

**5.C.II.** sSPN activity increased during the period when the animal made a choice whether or not to lick. This may suggest that sSPNs encode the action of licking.

**5.C.III.** sSPN subpopulations responded to the prediction error. Through the lens of the action model, perhaps sSPNs are revising the potential actions that will be initiated.

**5.C.IV.** sSPN activity was positively correlated with running velocity, and sSPN activity ramped along with licking bouts throughout the cue and outcome periods. This suggests that perhaps sSPNs encode the action of running.

**5.C.V.** sSPN subpopulations were most active when certain actions (e.g. turn direction) occurred, but between tasks these were not responsive during the same actions.

**Table 5:** Testing the alignment of the decision-space model to a selection of the experimental literature on GPi.

Criterion	Weglage et al. (2022)^73^ (I)	Munte et al. (2017)^78^ (II)	Stephenson-Jones et. al (2016)^79^ (III)
6.A	∅	✓	∅
6.B	∅	∅	✓
6.C	✓	∅	∅

✓ -- aligned with criterion

≈ -- somewhat aligned to criterion

✘ -- not aligned with criterion

∅ -- experiment does not test criterion

**6.A.II.** Level of reward correlated with GPi activity. This may suggest that GPi activity scales up or down depending on the level of information along a reward-related decision-dimension.

**6.B.III.** Individual LHb-projecting GPi neurons were both excited by punishment-predicting cues and the punishment itself and were inhibited by rewards and their associated cues. This may suggest that information along two decision-dimensions, one reward-related and one-cost related, is encoded by the same GPi neurons. Further, the opposite response of GPi neurons to reward and cost lends support for our choice to differentially weight GPi inputs from sSPN subpopulations corresponding to different decision-dimensions.

**6.C.I.** An identified subpopulation of LHb-projecting GPi affected the profile of choices made, aligning with the functional role of the GPi in our model. Decreased activity of these neurons was associated with increased commitment to actions, which may correspond to effective formation of a decision-space. This would align with the hypothesis of the model that lower GPi activity leads to a higher-dimensional decision-space.

**Table 6:** Testing the alignment of the decision-space model to a selection of the experimental literature on LHb and RMTg.

Criterion	Matsumoto & Hikosaka (2007)^80^ (I)	Lee & Hikosaka (2022)^81^ (II)	Stopper & Floresco (2014)^82^ (III)	Vento et al. (2017)^83^ (IV)
7.A	∅	∅	≈	✓
7.B	✓	✓	∅	∅

✓ -- aligned with criterion

≈ -- somewhat aligned to criterion

✘ -- not aligned with criterion

∅ -- experiment does not test criterion

**7.A.III.** LHb inactivation led subjects to change their choice during a probabilistic discounting task to accept a large, risky reward over a smaller, safe reward. As our model expects, LHb inactivation played an important role in affecting decisions that required multiple decision-dimensions. It is expected, however, that reduced LHb activity produces enhanced adherence to any decision-dimensions required to perform the task. The subjects with inactive LHb would appear to be incorporating fewer, not more, decision-dimensions into their choices. One possible explanation is that LHb inactivation led to an overwhelming increase in dimensionality of the decision-space that reduced focus on a few important decision-dimensions, such as reward.

**7.A.IV.** RMTg selectively altered decisions, primarily in response to cost. This may suggest that RMTg inactivation led to choices with less adherence to an important cost-related decision-dimension.

**7.B.I.** LHb activation led to suppression of dopaminergic signaling among daSNC neurons. LHb was active at times when it may not have been beneficial to construct a decision-space involving a reward-related decision-dimension (when no reward was presented) but inactive when it may have been beneficial to construct a decision-space using a reward-related decision-dimension (when reward was presented).

**7.B.II.** LHb was found to alter its activity depending on situational context. LHb was most active at times when it may not have been beneficial to construct a decision-space involving a reward-predominant decision-dimension (when it was indicated that minimal reward would be available or when less than expected reward was presented) and at times when evaluation of data along decision-dimensions may have been less necessary (the uncontrollable tasks).

**Table 7:** Testing the alignment of the decision-space model to a selection of the experimental literature on daSNC.

Criterion	Fiorillo et al. (2003)^84^ (I)	Matsumoto & Hikosaka (2009)^85^ (II)	Gan et al. (2010)^86^ (III)	Bromberg-Martin et al. (2010)^87^ (IV)	Kim et al. (2020)^88^ (V)	Long et al. (2024)^89^ (VI)
8.A	✓	✓	✓	✓	✓	∅
8.B	∅	∅	∅	∅	∅	≈

✓ -- aligned with criterion

≈ -- somewhat aligned to criterion

✘ -- not aligned with criterion

∅ -- experiment does not test criterion

**8.A.I.** daSNC neurons responded differently during the cue and during the outcome period depending on the likelihood of a cue predicting a reward outcome. This may suggest that a subpopulation of daSNC neurons encodes information along a reward-related decision-dimension, and probabilistic inputs are reflected continuously over time as the information along the decision-dimension is updated.

**8.A.II.** Two daSNC populations responded very differently to rewarding or aversive stimuli and a third group was non-responsive. This may support the tenet of the decision-space model that different daSNC subpopulations correspond to different decision-dimensions, some of which might be related to reward information, some to cost information, and some to neither reward nor cost information.

**8.A.III.** The activities of recorded dopamine neurons showed more resemblance to reward levels than to overall utility. This may support the tenet of the decision-space model that different dopamine subpopulations correspond to different decision-dimensions, some of which are related to reward information, and that dopamine neurons encode data along decision-dimensions, not an overall value function.

**8.A.IV.** Subpopulations of daSNC neurons that encoded value were excited by rewarding information while salience neurons were excited by both rewarding and aversive cues. This may support the tenet of the decision-space model that different daSNC subpopulations correspond to different decision-dimensions, some of which are related to reward information and some to other information.

**8.A.V.** Dopamine changed in response to altered proximity to reward. This may support the architecture of the decision-space model, where changes to reward information are captured in an sSPN subpopulation related to reward, then passed to a corresponding daSNC subpopulation.

**8.B.VI**. VTA cells were optogenetically inhibited or excited as ventral striatal neurons were recorded. Several findings are particularly relevant to the decision-space model: 1) A subpopulation of the striatal neurons responded to reward, and the activities of these neurons correlated with the VTA neurons. 2) 8% of all SPNs (4% above control, both non-reward-responding and reward-responding) had altered activities when VTA was inhibited. 3) The physical location of the SPNs that had altered activities had significantly distinct locations. Finding 1 may support the decision-space model, where mSPNs and sSPNs receive somewhat similar cortical inputs and sSPNs influence daSNC activity. A proportion of the reward-responding SPNs may encode a reward-related decision-dimension (the others may encode other information about the task and the reward administration). Further, per Finding 2, only a proportion of SPNs were affected by the reward-induced daSNC activity, supporting the selective release of dopamine to a reward-related SPN subpopulation in the model. Per Finding 3, the SPNs that did change their activities had spatial organization, supporting the assumption of our model that SPN subpopulations corresponding to decision-dimensions are organized spatially. The experiments, however, were primarily conducted on VTA neurons, not daSNC neurons, and a decoder did not accurately discriminate inhibition from control trials based on SPN spiking. This might be because a reward-related decision-space would require the modulation of more than 8% of neurons. Per our model, a decision-space would be formed in cases where sufficient dopamine was released to modulate a larger percentage (but not all SPNs). Indeed, when VTA neurons were manipulated to release more dopamine than they ordinarily did during the head-fixed licking task, up to 37% SPNs responded and a decoder successfully linked firing rates to the task, suggesting the formation of a decision-space. It may be that a decision-space only forms in certain tasks, for instance perhaps in tasks that require action selection, a hypothesis that might be supported by the findings of Samejima et al.^90^ and Seo et al.^91^ Our model provides a framework for task-dependent dopamine release to be studied in future work.

**Table 8:** Interpreting a selection of the experimental literature on the direct versus indirect pathways through the decision-space model.

Criterion	Parker et al. (2018)^92^ (I)	Peak et al. (2020)^93^ (II)	Maltese et al. (2021)^43^ (III)	Barbera et al. (2016)^27^ (IV)
9.A	✓	∅	✓	✓
9.B	∅	✓	∅	✓
9.C	∅	∅	∅	✓

✓ -- aligned with criterion

≈ -- somewhat aligned to criterion

✘ -- not aligned with criterion

∅ -- experiment does not test criterion

**9.A.I.** The direct and indirect pathway were found to be typically coactivated, and dopamine depletion differentially altered direct versus indirect pathway dynamics. This may support the tenet of our model that a decision-space is formed in each pathway during a decision and the two decision-spaces are affected differently by dopamine.

**9.A.III.** Increased dopamine release led to increased activation of dSPNs and decreased activation of iSPNs. Decreased dopamine release led to the opposite change. This supports the decision-space model, in which dopamine excites dmSPNs and inhibits imSPNs.

**9.A.IV.** Cocaine led to the increase in activity of a direct pathway subpopulation and simultaneously a decrease in activity of a neighboring indirect pathway subpopulation. This may suggest the simultaneous activation of a direct pathway decision-dimension and inactivation of a direct pathway decision-dimension.

**9.B.II.** Inhibition of dSPNs during learning led to blunted action associations, while inhibition of iSPNs led to a reduced ability to switch actions based on context. This supports the decision-space model, in which the direct pathway is involved with performing actions and the indirect pathway is involved in refraining from actions.

**9.B.IV.** Cocaine administration led to increased direct pathway activity, decreased indirect pathway activity, and more movement. This may suggest that the direct pathway more than the indirect pathway is associated with initiating actions.

**9.C.IV.** Subpopulations encoding data along decision-dimensions showed high intra-cluster synchrony that was stable across days, and inter-cluster synchrony was significantly lower. This may support the tenet of the decision-space model that similar information is encoded within proximate SPN subpopulations.
